# Utility of atherosclerosis-associated serum antibodies against colony-stimulating factor 2 in predicting the onset of acute ischemic stroke and prognosis of colorectal cancer

**DOI:** 10.3389/fcvm.2023.1042272

**Published:** 2023-02-10

**Authors:** Shu-Yang Li, Yoichi Yoshida, Masaaki Kubota, Bo-Shi Zhang, Tomoo Matsutani, Masaaki Ito, Satoshi Yajima, Kimihiko Yoshida, Seiichiro Mine, Toshio Machida, Aiko Hayashi, Minoru Takemoto, Koutaro Yokote, Mikiko Ohno, Eiichiro Nishi, Kenichiro Kitamura, Ikuo Kamitsukasa, Hirotaka Takizawa, Mizuki Sata, Kazumasa Yamagishi, Hiroyasu Iso, Norie Sawada, Shoichiro Tsugane, Katsuro Iwase, Hideaki Shimada, Yasuo Iwadate, Takaki Hiwasa

**Affiliations:** ^1^Department of Neurological Surgery, Graduate School of Medicine, Chiba University, Chiba, Japan; ^2^Department of Biochemistry and Genetics, Graduate School of Medicine, Chiba University, Chiba, Japan; ^3^Comprehensive Stroke Center, Chiba University Hospital, Chiba, Japan; ^4^Department of Clinical Oncology, Toho University Graduate School of Medicine, Tokyo, Japan; ^5^Department of Gastroenterological Surgery, Toho University Graduate School of Medicine, Tokyo, Japan; ^6^Department of Neurological Surgery, Chiba Prefectural Sawara Hospital, Chiba, Japan; ^7^Department of Neurological Surgery, Chiba Cerebral and Cardiovascular Center, Chiba, Japan; ^8^Department of Neurosurgery, Eastern Chiba Medical Center, Chiba, Japan; ^9^Department of Endocrinology, Hematology and Gerontology, Graduate School of Medicine, Chiba University, Chiba, Japan; ^10^Department of Diabetes, Metabolism and Endocrinology, School of Medicine, International University of Health and Welfare, Chiba, Japan; ^11^Department of Cardiovascular Medicine, Graduate School of Medicine, Kyoto University, Kyoto, Japan; ^12^Department of Pharmacology, Shiga University of Medical Science, Otsu, Shiga, Japan; ^13^Kitakurihama Takuchi Clinic, Yokosuka, Kanagawa, Japan; ^14^Department of Neurology, Chiba Rosai Hospital, Chiba, Japan; ^15^Port Square Kashiwado Clinic, Kashiwado Memorial Foundation, Chiba, Japan; ^16^Department of Public Health Medicine, Faculty of Medicine, and Health Services Research and Development Center, University of Tsukuba, Tsukuba, Japan; ^17^Department of Preventive Medicine and Public Health, Keio University School of Medicine, Tokyo, Japan; ^18^Public Health, Department of Social Medicine, Osaka University Graduate School of Medicine, Suita, Japan; ^19^Division of Cohort Research, National Cancer Center Institute for Cancer Control, Tokyo, Japan

**Keywords:** colony-stimulating factor 2, acute ischemic stroke, colorectal cancer, antibody biomarker, atherosclerosis

## Abstract

**Introduction:**

Autoantibodies against inflammatory cytokines may be used for the prevention of atherosclerosis. Preclinical studies consider colony-stimulating factor 2 (CSF2) as an essential cytokine with a causal relationship to atherosclerosis and cancer. We examined the serum anti-CSF2 antibody levels in patients with atherosclerosis or solid cancer.

**Methods:**

We measured the serum anti-CSF2 antibody levels *via* amplified luminescent proximity homogeneous assay-linked immunosorbent assay based on the recognition of recombinant glutathione S-transferase-fused CSF2 protein or a CSF2-derived peptide as the antigen.

**Results:**

The serum anti-CSF2 antibody (s-CSF2-Ab) levels were significantly higher in patients with acute ischemic stroke (AIS), acute myocardial infarction (AMI), diabetes mellitus (DM), and chronic kidney disease (CKD) compared with healthy donors (HDs). In addition, the s-CSF2-Ab levels were associated with intima-media thickness and hypertension. The analyzes of samples obtained from a Japan Public Health Center-based prospective study suggested the utility of s-CSF2-Ab as a risk factor for AIS. Furthermore, the s-CSF2-Ab levels were higher in patients with esophageal, colorectal, gastric, and lung cancer than in HDs but not in those with mammary cancer. In addition, the s-CSF2-Ab levels were associated with unfavorable postoperative prognosis in colorectal cancer (CRC). In CRC, the s-CSF2-Ab levels were more closely associated with poor prognosis in patients with p53-Ab-negative CRC despite the lack of significant association of the anti-p53 antibody (p53-Ab) levels with the overall survival.

**Conclusion:**

S-CSF2-Ab was useful for the diagnosis of atherosclerosis-related AIS, AMI, DM, and CKD and could discriminate poor prognosis, especially in p53-Ab-negative CRC.

## 1. Introduction

Cytokines, such as interleukins, tumor necrosis factor, interferons, and colony-stimulating factors, contribute to the presence and development of a variety of diseases (1, 2), including cancer (3), acute ischemic stroke (AIS; 4), and acute myocardial infarction (AMI; 5). As a cytokine, colony-stimulating factor 2 (CSF2, also known as granulocyte-macrophage colony-stimulating factor) deficiency has been reported to be associated with increased atherogenesis under hypercholesterolemic conditions in mouse models (6), and the administration of CSF2 can prevent the progression of atherosclerosis through changes in the composition of atherosclerotic lesions (7). Atherosclerosis is intimately linked to and accompanied by diabetes mellitus (DM) and chronic kidney disease (CKD) (8). Several reports have demonstrated that atherosclerosis mainly contributes to the emergence of AIS and AMI (9, 10).

Intriguingly, growing evidence supports that atherosclerosis and cancer are closely related based on the shared pathophysiology of inflammation as a disease promoter (11, 12). Furthermore, CSF2 has been shown to exert antitumor activity in gastrointestinal tract cancers, such as esophageal cancer (EC; 13), colorectal cancer (CRC) (14), and gastric cancer (GC; 15). CSF2 upregulation was associated with increased aggressiveness of various tumor types, including head and neck cancers (16), glioblastomas (17), and bladder cancer (18). The utility of widely used serum biomarkers, such as carcinoembryonic antigen (CEA) (19), carbohydrate antigen 19–9 (CA19-9) (20), and anti-p53 antibody (p53-Ab; 21–23), for the early diagnosis and prognosis prediction of gastrointestinal tract cancers is limited (24, 25). The discovery of novel biomarkers in gastrointestinal tract cancers is required for the prognostic prediction and development of targeted therapeutics.

Recent studies have reported the production of serum autoantibodies against secreted proteins (25–32). Autoantibodies produced against cytokines circulating in the peripheral blood can potentially impact their binding to target membrane receptors and the subsequent disease progression. We have previously identified autoantibodies against CSF2 in the sera of patients with acute cardiac syndrome (33). In this study, we examined the serum anti-CSF2 antibody levels in patients with AIS, AMI, DM, CKD, and cancer.

## 2. Materials and methods

### 2.1. Selection of patients and healthy donors

The sera of patients with AIS and transient ischemic attack (TIA), which were collected within 2 weeks after disease onset, were obtained from Chiba Prefectural Sawara Hospital and Chiba Rosai Hospital. The stroke subtypes were determined according to the criteria of the Trial of ORG 10172 in Acute Stroke Treatment classification system (34), and large-artery atherosclerosis and small-arterial occlusion (lacuna) were included as AIS or ischemic stroke. The sera of patients with DM and AMI were obtained from Chiba University Hospital and Kyoto University Hospital, respectively. The sera of patients with CKD were obtained from the Kumamoto cohort (35, 36). The Department of Surgery, Toho University Hospital, collected sera from patients with EC, GC, CRC, lung cancer (LC), and mammary cancer (MC; 37) between June 2010 and February 2016, and all patients were followed up until July 2018 or death. EC was analyzed in 91 cases, GC was analyzed in 57 cases and CRC was analyzed in 113 cases, of which all cases were underwent radical surgery. Patients who underwent neoadjuvant chemotherapy and had a double cancer were excluded from the study. According to the Japanese Classification of Colorectal, Appendiceal, and Anal Carcinoma, 3d English Edition (Secondary Publication; 38), the numbers of patients with colorectal cancer were as follows: five patients in stage 0, 29 in stage I, 32 in stage II, 31 in stage III, and 16 in stage IV. In addition, the sera of healthy donors (HDs) were selected from people who underwent a medical checkup at Chiba University, Port Square Kashiwado Clinic, and Chiba Prefectural Sawara Hospital. Clinicopathological characteristics and prognoses were obtained retrospectively. Individuals with no history of cancer, autoimmune disease, or cerebrovascular disease and those without any abnormalities on cranial MRI were enrolled as HDs.

Serum samples of AIS, TIA, DM, CKD, and HD were collected at the time of hospital admission. Serum samples of the cancers were collected before treatment. All serum samples were centrifuged at 3,000 g for 10 min, and the supernatants were stored at −80°C until use. To preserve sample integrity, repeated freezing/thawing of serum samples was avoided.

### 2.2. Clinical data

Clinical data of all patients; e.g., maximum intima-media thickness (max-IMT; 39), were the maximum values of the intima-media thickness for both left and right sides of the carotid ultrasound results performed using a high-frequency line array probe. The plaque score was computed by summing the maximum thickness (millimeters) of the plaques in each segment on both sides (40, 41). The cardio-ankle vascular index (CAVI; 42), which is independent of blood pressure, was developed using CAVI = a{(2ρ/ΔP) x ln(Ps/Pd)PWV2} + b, where Ps is the systolic blood pressure, Pd is the diastolic blood pressure, PWV is the pulse wave velocity, ΔP is Ps–Pd, ρ is blood density, and a and b are scale conversion constants. Scale conversion constants were determined to match CAVI with PWV (Hasegawa’s method). All these measurements and calculations were made at the same time automatically in VaSera (Fukuda Denshi Co. LTD, Tokyo). All data were collected by Japanese clinical professionals and were written in the patient’s clinical record. Data regarding the risk factors for atherosclerosis, including hypertension, diabetes, hyperlipidemia, cardiovascular disease (CVD), smoking, and alcohol usage were defined as previously described (43, 44) and the levels of serum p53 antibodies (p53-Abs; 22), serum CEA (45) and CA19-9 (46) were also evaluated as previously described. The cutoff values for serum p53-Ab, CEA, and CA19-9 levels were set at 1.3 IU/ml, 10 ng/ml, and 37.0 ng/ml, respectively.

### 2.3. Expression and purification of CSF2 protein

The cDNA containing the amino-terminal (first 145 amino-acid residues) of CSF2 (NM_000758.4) was inserted into the *Bam*HI*/Not*I site of pGEX-4 T-1 to obtain the pGEX-4 T-1-CSF2 plasmid (GenScript, Piscataway, NJ). The expression of the cDNA product was induced by treating pGEX-4 T-1-CSF2-transformed *Escherichia coli* BL-21 with 0.1-mM isopropyl-β-D-thiogalactoside (Wako Pure Chemicals, Osaka, Japan) for 4 h at 37°C. The cells were subsequently lysed in BugBuster Master Mix (Merck KGaA, Darmstadt, Germany). Glutathione S-transferase (GST)-tagged CSF2 protein was purified using Glutathione-Sepharose® (Cytiva, Pittsburgh, PA column chromatography according to the manufacturer’s protocols, as previously described) (43, 47).

### 2.4. Generation of the CSF2 peptide antigen

The potential antigenic epitopes in CSF2 were predicted through the examination of the full-length CSF2 protein using an online tool^1^, as previously described (48, 49). An *N*-terminal biotinylated 18-mer peptide encompassing the amino-acid residues between 77 and 94 of CSF2 was designed (33), and the synthetic peptide with a purity >95.93% was manufactured by Eurofins Genomics K.K. (Tokyo, Japan). The amino-acid sequence of the peptide, designated as bCSF2-77, is as follows: biotin-LYKQGLRGSLTKLKGPLT-COOH.

### 2.5. Western blotting

Purified GST-CSF2 and control GST proteins were electrophoresed using 11% sodium dodecyl-sulfate (SDS)–polyacrylamide gels, followed by transfer to nitrocellulose membranes (Advantec, Tokyo, Japan). The membranes were blocked with 0.1% dry milk in Tris-buffered saline [150 mM NaCl, 20 mM Tris–HCl (pH 7.6), and 0.1% Tween-20; TBS-T] and subsequently incubated with anti-GST antibodies (goat, ab6613, Abcam, Cambridgeshire, United Kingdom), anti-CSF2 antibodies (rabbit, sc-37,753, Santa Cruz Biotechnology, Dallas, TX), or HD and patient sera diluted at 1:1000. Thereafter, the membranes were washed five times with TBS-T and incubated with horseradish peroxidase-conjugated second antibodies (anti-goat IgG, anti-rabbit IgG, and antihuman IgG; Santa Cruz Biotechnology, Santa Cruz, CA) for 20 min. After washing with TBS-T five times, Immobilon Western Chemiluminescent Substrate (Merck KGaA) was added to the membranes, and luminescence was detected using LuminoGraph II (Atto, Tokyo, Japan), as previously described (30, 44, 50).

### 2.6. Measurement of serum anti-CSF2 and anti-bCSF2-77 peptide antibody levels

Amplified luminescence proximity homogeneous assay-linked immunosorbent assay (AlphaLISA) was employed to measure the serum anti-CSF2 antibody (s-CSF2-Ab) and serum anti-bCSF2-77-peptide antibody (s-CSF2pep-Ab) levels. The reaction mixture containing 2.5 μL of serum samples diluted at 1:100 in AlphaLISA buffer (25 mM HEPES pH 7.4, 0.1% casein, 0.5% Triton X-100, 1 mg/mL of dextran-500, and 0.05% Proclin-300) and 2.5 μL of GST or GST-CSF2 proteins (10 μg/ml) or 400 ng/mL of bCSF2-77 was incubated in 384-well white opaque OptiPlate microtiter plates (PerkinElmer, Beaconsfield, United Kingdom) at room temperature for 6–8 h. Next, 2.5 μL of anti-human immunoglobulin G (IgG)-conjugated acceptor beads (40 μg/ml) and 2.5-μL glutathione-conjugated donor beads (40 μg/ml) or 2.5 μL of streptavidin-conjugated donor beads (40 μg/ml) were added. After incubation in the dark for 7–28 days at room temperature, chemical emission was measured using an EnSpire Alpha microplate reader (PerkinElmer), as previously described (48, 51, 52). Specific reactions were calculated by subtracting the emitted alpha photon counts of the GST and buffer controls from those of the GST-CSF2 protein and the bCSF2-77 peptide, respectively.

### 2.7. Japan public health center cohort analysis

The longitudinal correlation between plasma s-CSF2pep-Ab levels and AIS in the Japan Public Health Center (JPHC)-based prospective study was investigated using AlphaLISA. The study nested within the JPHC cohort (53, 54) included approximately 30,000 Japanese individuals aged 40–69 years at the baseline period of 1990–1994 with stored plasma samples. The s-CSF2pep-Ab levels were measured in 202 cases who were included in the cohort between the baseline period and 2008 and in 202 controls whose sex, age (within 2 years), date of blood sampling (within 3 months), time since the last meal (within 4 h), and study location were matched with the cases. We used conditional logistic regression model to estimate the odds ratios (ORs) with 95% confidence intervals (CIs) for estimating the risk of AIS based on s-CSF2pep-Ab levels.

### 2.8. Cell culture

LoVo cells, a human CRC line expressing wild-type p53 (55), and DLD1 cells, a human CRC line expressing mutant p53 (56), were cultured at 37°C in a humidified atmosphere containing 5% CO_2_. LoVo cells were cultured in Ham’s F-12 (Ham’s F-12, NACALAI TESQUE, Inc., Kyoto, Japan) supplemented with 10% heat-inactivated fetal bovine serum (Thermo Fisher Scientific, Waltham, MA) and 100 μg/ml of kanamycin (Meiji Pharmaceutical, Kyoto, Japan). DLD1 cells were cultured in Dulbecco’s Modified Eagle’s Medium (NACALAI TESQUE) and supplemented with 10% fetal bovine serum and 100 μg/ml of kanamycin.

### 2.9. MTS assay

The effects of CSF2 and s-CSF2-Ab on the proliferation and viability of LoVo and DLD1 cells were evaluated using 3-(4,5-dimethylthiazol-2-yl)-5-(3-carboxymethoxyphenyl)-2-(4-sulfophenyl)-2H-tetrazolium (MTS, Promega, Madison, WI) assay, as previously reported (57, 58). Briefly, the cells were seeded into 96-well plates at a density of 2 × 10^3^ cells/well. Next, rabbit anti-CSF2 antibody (10 μg/ml; Cusabio Technology, Houston, TX), CSF2 (1 μg/ml; PeproTech, Rocky Hill, NJ), or anti-CSF2 antibody (10 μg/ml) plus CSF2 (1 μg/ml) was added, and the plates were incubated for 72 h at 37°C in a humidified atmosphere containing 5% CO_2_. For the MTS assay, 20 μl of the 20:1 MTS (1.9 mg/ml)-PMS (phenazine methosulfate, Promega; 44 μg/ml) dye solution (CellTiter 96 AQueous Nonradioactive Cell Proliferation Assay, Promega) was directly added to each well, and the cells were incubated for 3–4 h. The absorbance at 490 nm was measured using a microplate reader (Emax, Molecular Devices, Sunnyvale, CA), and the data were expressed as relative cell viability ratios, i.e., absorbance of each experimental group versus the drug-free control.

### 2.10. Luciferase reporter assay

LoVo cells (1 × 10^5^ cells/well) seeded into 24-well plates were transfected with pCMV-p53WT plasmid (0.5 μg), a plasmid expressing wild-type p53, together with pGL3-p21-Luc (0.1 μg), a firefly luciferase reporter plasmid containing the promoter region of *p21*/*Cip1*, and pRL-SV40 (0.01 μg; Promega), a control *Renilla* luciferase reporter plasmid, using Lipofectamine Plus (Thermo Fisher Scientific). pCMV-p53WT was generously provided by Dr. Bert Vogelstein (Howard Hughes Medical Institute), and pGL3-p21-Luc (59) was kindly provided by Dr. Mian Wu (University of Science and Technology of China; 60). After 24 h of incubation, ultrapure water, rabbit anti-CSF2 antibody (10 μg/ml), or CSF2 (1 μg/ml) was added to each well. Two days after the transfection, firefly and *Renilla* luciferase activities were determined using the Dual-Luciferase Assay System (Promega) and a luminescence reader (Atto, Tokyo, Japan), as previously reported (49, 60). The firefly luciferase activity was normalized to the luciferase activity of the *Renilla* control plasmid.

### 2.11. Analysis of phosphorylated p53 levels

LoVo cells were treated with 1 μg/ml of CSF2 or 10 μg/ml of anti-CSF2 antibody for 4 h, followed by washing with phosphate-buffered saline. The cells were lysed in 2% SDS, 10% glycerol, 50-mM Tris-Cl (pH 6.8), 0.01% bromophenol blue, and 1% β-mercaptoethanol. The lysates were incubated at 100°C for 3 min, sonicated for 10 min, separated on 9% SDS–polyacrylamide gels, and transferred to nitrocellulose membranes. The following primary antibodies were used with 1:1000 dilution in TBS-T: rabbit anti-Ser-46 phospho-specific p53 antibody (sc-101,764, Santa Cruz Biotechnology), rabbit anti-Ser-392 phospho-specific p53 antibody (sc-7,997-R, Santa Cruz Biotechnology), mouse anti-Ser-315 phospho-specific p53 antibody (K0059-3, Medical & Biological Laboratories, Nagoya, Japan), rabbit anti-Thr-81 phospho-p53 antibody (2,676, Cell Signaling Technology, Danvers, MA), and rabbit anti-β-actin antibody (sc-8,432, Santa Cruz Biotechnology).

### 2.12. Statistical analysis

The Mann–Whitney *U* test and Student’s *t*-test (two-sided) were employed to determine significant differences between the two groups, and the Kruskal–Wallis test (Mann–Whitney *U* with Bonferroni’s correction applied) was employed to evaluate the differences among three or more groups. Correlations were determined using Spearman’s correlation and logistic regression analyzes. All statistical analyzes were conducted using GraphPad Prism 5 (GraphPad, San Diego, CA) and the EZR software (61). Western blotting analysis was conducted using the ImageJ software (NIH, Bethesda, MD). A repeated-measured analysis of variance (ANOVA) with Tukey’s *post hoc* comparisons was performed on the MTS assay results and luciferase reporter assay results. The predictive values of the putative disease markers were evaluated using receiver operating characteristic (ROC) curve analysis, and the cutoff values were set to maximize the sums of sensitivity plus specificity. Furthermore, the analyzes of patient survival were evaluated using the Kaplan–Meier method and compared using the log-rank test. All tests were two-tailed, and *P* values <0.05 were considered to indicate statistically significant differences.

## 3. Results

### 3.1. Presence of serum antibodies against purified proteins in patients with AIS, DM, EC, and CRC

**Figure 1:**
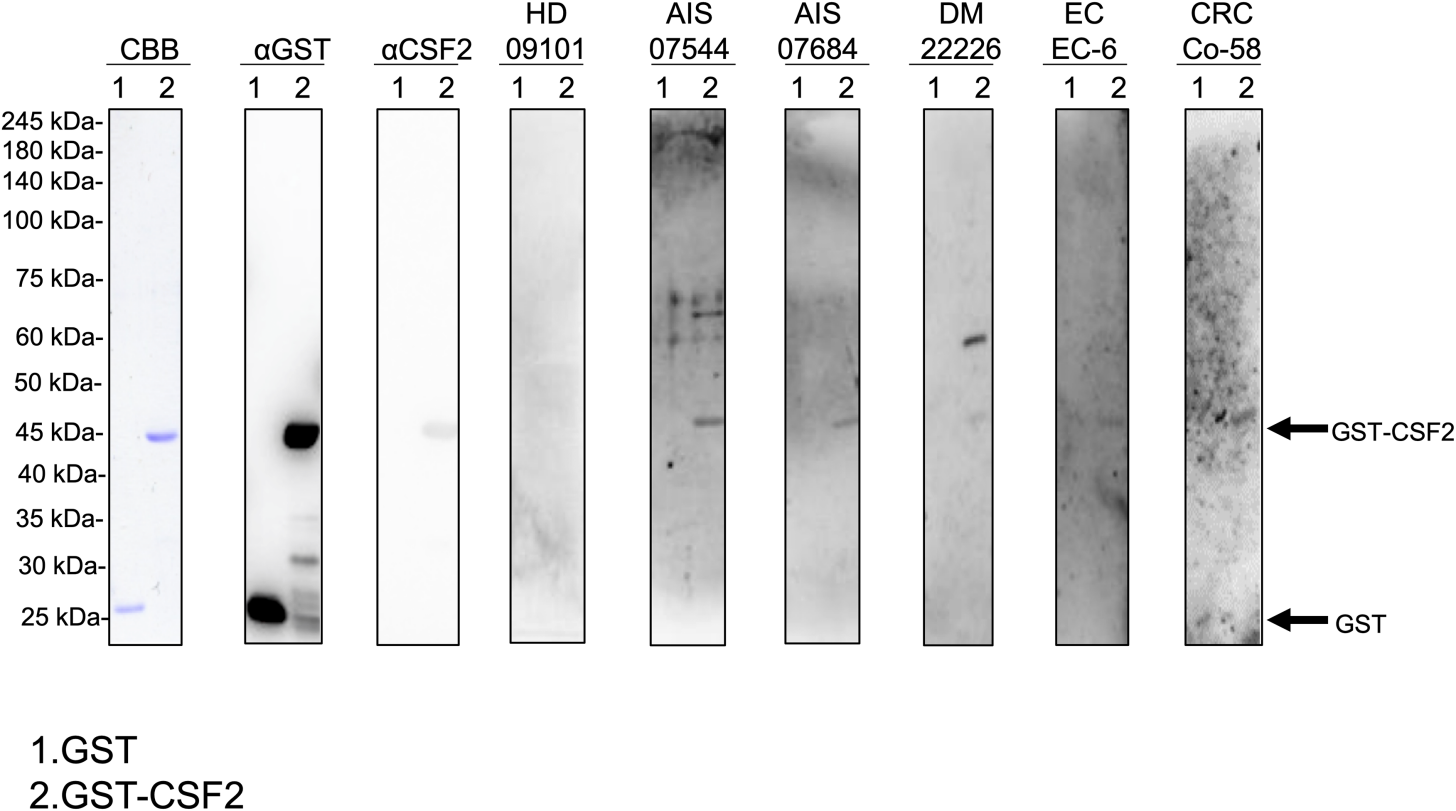
Detection of serum antibodies against CSF2. Presence of antibodies against CSF2 in the serum samples of patients with acute ischemic stroke (AIS), diabetes mellitus (DM), esophageal cancer (EC), and colorectal cancer (CRC). Purified GST-CSF2 (lane 1) and GST (lane 2) proteins were separated on sodium dodecyl sulfate–polyacrylamide gels, followed by staining with Coomassie Brilliant Blue (CBB) or western blotting using anti-GST (αGST), anti-CSF2 (αCSF2), and serum samples from patients with AIS (07544, 07684), DM (22226), EC (EC-6), and CRC (Co-58) and from a healthy donor (HD)(09101). The full, non-adjusted image of Figure 1 is shown in the Supplementary Figure S1. Molecular weights are presented in the left lane. Arrows indicate the positions of GST-CSF2 (44.6 kDa) and GST (26 kDa). CSF2, colony-stimulating factor 2.

We performed western blotting to confirm the presence of serum autoantibodies against CSF2 in the sera of patients with AIS, DM, EC, and CRC. As presented in Figure 1, the recombinant GST-CSF2 protein reacted with the commercial anti-GST and anti-CSF2 antibodies, whereas the control GST protein reacted with the anti-GST but not with the anti-CSF2 antibody. The GST-CSF2 protein was also recognized by serum IgG antibodies from patients with AIS (#07544 and #07684), DM (#22226), EC (#EC-6), and CRC (#Co-58) but not by the sera of the HD (#09101). To avoid contingency, we performed western blotting for more serum samples. The GST-CSF2 protein was also recognized by serum IgG antibodies from patients with AIS (#007303, #07344, and #07684), TIA (#02291, #07278, and #07642), DM (#22297, #22370, and #22375), EC (#EC-3, #EC-4, and #EC-6), and CRC (#Co-3, #Co-5, and #Co-12), but not by the sera of the HDs (#09101, #07547, and #07572; Supplementary Figure S2). And the GST protein alone did not demonstrate observable reactivity with any of these serum samples (data not shown).

### 3.2. The s-CSF2-Ab and s-CSF2pep-Ab levels are elevated In patients with AIS and TIA

**Figure 2:**
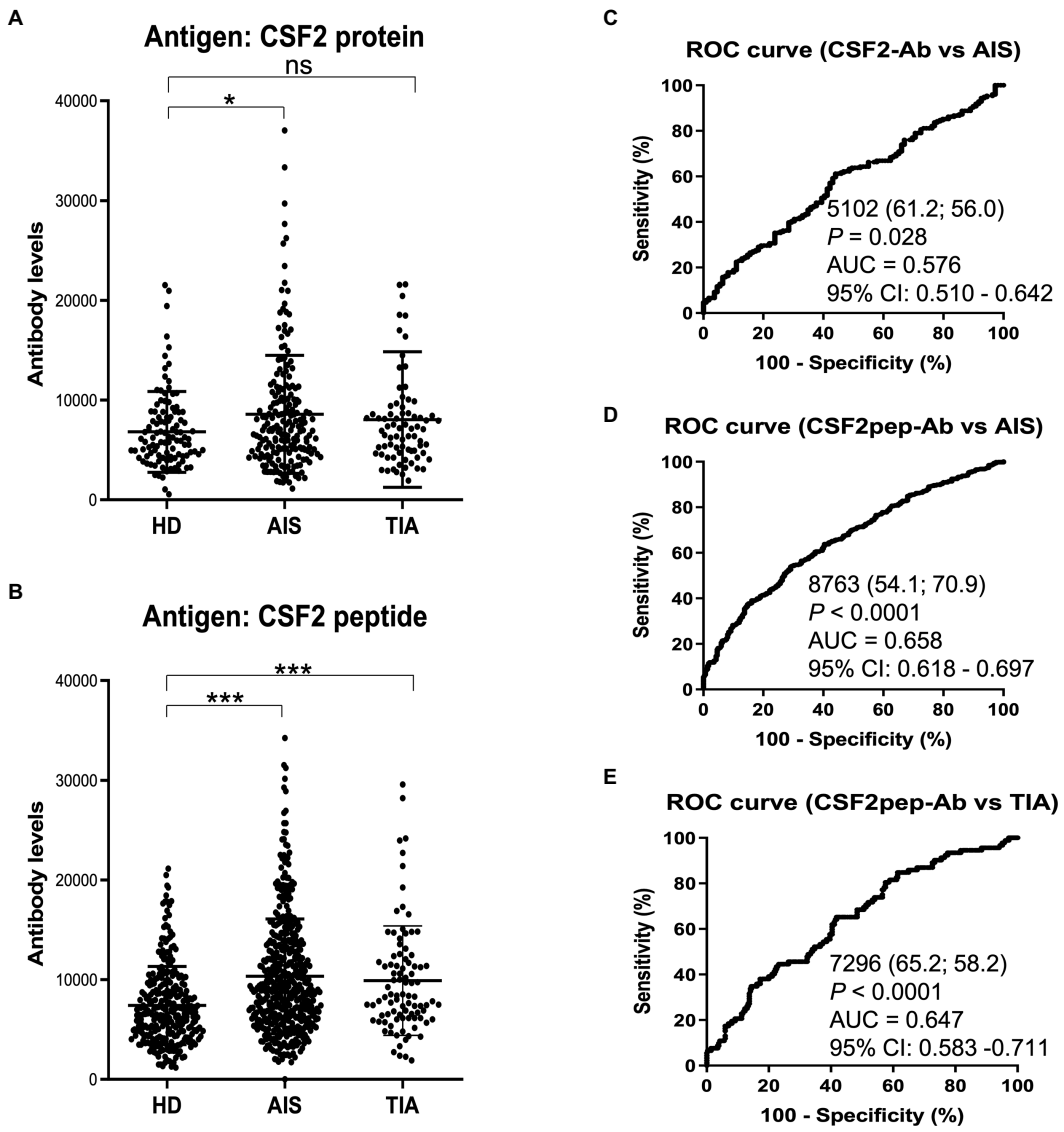
Comparison of the s-CSF2-Ab and s-CSF2pep-Ab levels in patients with AIS and TIA and in HDs. **(A–B)** Levels of serum antibodies against **(A)** s-CSF2-Ab and **(B)** s-CSF2pep-Ab determined using amplified luminescence proximity homogeneous assay-linked immunosorbent assay (AlphaLISA), calculated by subtracting the levels of antibodies against the control GST. Scatter dot plots of the s-CSF2-Ab and s-CSF2pep-Ab levels are also presented. Bars represent averages ± standard deviation (SD). **P < 0.05; *P < 0.01; ns, not significant, Kruskal–Wallis test. **(C–E)** A receiver operating characteristic (ROC) curve analysis was conducted to assess the ability of s-CSF2-Ab to detect AIS **(C)** and that of s-CSF2pep-Ab to detect AIS **(D)** and TIA **(E)**. The numbers indicate the cutoff values for the indicated markers, and the numbers in parentheses indicate sensitivity (left) and specificity (right). The areas under the ROC curve (AUC) and 95% confidence intervals (CIs) are also shown. AIS, acute ischemic stroke; HD, healthy donor; CSF2-Ab, CSF2 antibody; CSF2pep-Ab, CSF2 peptide antibody; TIA, transient ischemic attack.

Next, we examined the s-CSF2-Ab levels in HDs and patients with AIS and TIA using AlphaLISA. As presented in Figure 2A, the s-CSF2-Ab levels were significantly higher in patients with AIS than in HDs (Figure 2A). Similarly, the s-CSF2pep-Ab levels were significantly higher in patients with AIS and TIA than in HDs (Figure 2B). Based on the cutoff s-CSF2-Ab level, defined as two standard deviations (SDs) above the average s-CSF2-Ab level in HDs, the s-CSF2-Ab positivity rates were 5.5% in HDs and 12.2% in patients with AIS (Table 1). Using the same approach to define the cutoff s-CSF2pep-Ab level, the rates of s-CSF2pep-Ab positivity were 5.6, 18.1, and 10.9% in HDs and in patients with AIS and TIA, respectively. Furthermore, the ROC analysis revealed that the areas under the curve (AUCs) for s-CSF2-Ab vs. AIS, s-CSF2pep-Ab vs. AIS, and s-CSF2pep-Ab vs. TIA were 0.576, 0.658, and 0.647, respectively (Figures 2C–E), suggesting that the s-CSF2pep-Ab level was more closely associated with AIS than the s-CSF2-Ab level.

**Table 1:** Comparison of the s-CSF2-Ab and s-CSF2pep-Ab levels in HDs versus those in patients with AIS and TIA.

Sample information		HD	AIS	TIA
Total sample number		109	196	79
Male/Female		62/47	122/74	46/33
Age (Average ± SD)		59.82 ± 7.90	75.13 ± 7.28	70.67 ± 12.75				
		s-CSF2-Ab	s-CSF2pep-Ab	
HD	Average	5,944	7,406	
	SD	4,315	3,929	
	Cutoff values	14,575	15,264	
	Total number	109	285	
	Positive number	6	16	
	Positive (%)	5.5%	5.6%	
AIS	Average	7,753	10,334	
	SD	6,299	5,764	
	Total number	196	464	
	Positive number	24	84	
	Positive (%)	**12.2%**	**18.1%**	
	*P* value (*vs* HD)	**<0.05**	**<0.0001**	
TIA	Average	6,980	9,903	
	SD	7,680	5,477	
	Total number	79	92	
	Positive number	7	10	
	Positive (%)	8.9%	**10.9%**	
	*P* value (*vs* HD)	ns	**<0.0001**	

The upper panel indicates the number of total samples, samples from male and female participants, and ages [average ± standard deviation (SD)]. The lower panel summarizes the serum antibody levels examined by amplified luminescence proximity homogeneous assay-linked immunosorbent assay (AlphaLISA). Purified CSF2-GST proteins and CSF2 peptides were used as antigens. The cutoff values were determined as the average HD values plus two SDs, and positive samples for which the Alpha counts exceeded the cutoff value were scored. *P* values were calculated using the Kruskal–Wallis test (Mann–Whitney U test with Bonferroni’s correction applied). *P* values lower than 0.05 and positive rates higher than 10% are marked in bold. These data are plotted and shown in Figures 2A,B. CSF2, colony-stimulating factor 2; s-CSF2-Abs, the serum anti-CSF2 antibodies; s-CSF2pep-Abs, the serum anti-CSF2 peptide antibodies; HDs, healthy donors; AIS, acute ischemic stroke; TIA, transient ischemic attack.

### 3.3. The s-CSF2-Ab and s-CSF2pep-Ab levels are elevated in patients with AMI and DM

**Figure 3:**
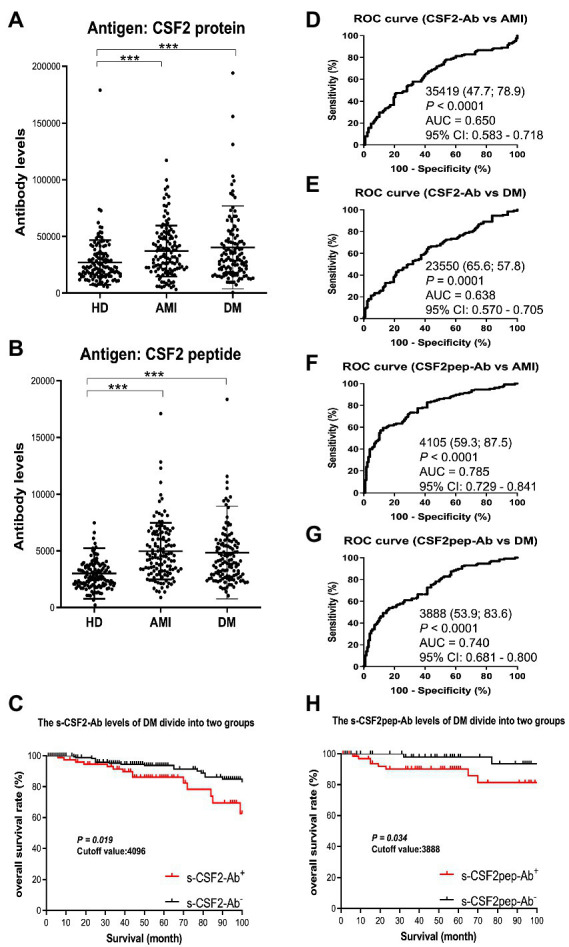
Comparison of the s-CSF2-Ab and s-CSF2pep-Ab levels between HDs and patients with AMI and DM. (**A,B**) The levels of s-CSF2-Ab and s-CSF2pep-Ab in HDs and patients with acute myocardial infarction (AMI) (**A**) and diabetes mellitus (DM) (**B**) were determined using AlphaLISA. The bars represent averages ± SD. *** *P* < 0.001, Mann–Whitney *U* test. (**D,E**) The ROC curves to assess the ability of s-CSF2-Ab to predict AMI and DM are presented in (**D**) and (**E**), respectively. (**F,G**) The ability of s-CSF2pep-Ab to predict AMI (**E**) and DM (**F**) was also evaluated *via* ROC analysis. Comparison of the overall survival in patients with DM between the s-CSF2-Ab positive (s-CSF2-Ab^+^) and s-CSF2-Ab negative (s-CSF2-Ab^−^) groups (*P* = 0.019; **C**) and between the s-CSF2pep-Ab positive (s-CSF2-Ab^+^) and s-CSF2pep-Ab negative (s-CSF2-Ab^−^) groups (*P* = 0.034; **C**). Statistical analyzes were performed by the Log-Rank test between two groups.

Next, we measured the s-CSF2-Ab and s-CSF2pep-Ab levels in patients with AMI and DM. The average (±SD) ages of HDs and patients with AMI and DM were 58.29 ± 5.63, 58.20 ± 8.50, and 58.48 ± 9.17 years, respectively (Table 2). The s-CSF2-Ab and s-CSF2pep-Ab levels were significantly higher in patients with AMI and DM than in HDs (Figures 3A,B). Based on the cutoff s-CSF2-Ab and s-CSF2pep-Ab levels, defined as two SDs above the average levels in HDs, the rates of s-CSF2-Ab and s-CSF2pep-Ab positivity were 2.3 and 1.6% in HDs, 10.2 and 11.7% in patients with AMI, and 13.3 and 10.2% in patients with DM, respectively (Table 2). The ROC analysis revealed that the AUCs for s-CSF2-Ab were 0.650 and 0.638 for AMI and DM, respectively. Similarly, the ROC analysis revealed that the AUCs for s-CSF2pep-Ab were 0.785 and 0.740 for AMI and DM, respectively. The higher AUCs for both s-CSF2-Ab and s-CSF2pep-Ab in AMI and DM than in AIS and TIA (Figures 3D–G) suggested that s-CSF2-Ab and s-CSF2pep-Ab might be more closely associated with AMI and DM than with AIS and TIA.

**Table 2:** Comparing the s-CSF2-Ab and s-CSF2pep-Ab levels between HDs and patients with AMI or DM.

Sample information		HD	AMI	DM
Total sample number		128	128	128
Male/Female		72/56	105/23	72/56
Age (Average ± SD)		58.29 ± 5.63	58.20 ± 8.50	58.48 ± 9.17				
		s-CSF2-Ab	s-CSF2pep-Ab	
HD	Average	27,010	3,006	
	SD	19,725	2,234	
	Cutoff values	66,460	7,474	
	Total number	128	128	
	Positive number	3	2	
	Positive (%)	2.3%	1.6%	
AMI	Average	37,075	4,979	
	SD	22,495	2,503	
	Total number	128	128	
	Positive number	13	15	
	Positive (%)	**10.2%**	**11.7%**	
	*P* value (*vs* HD)	**<0.0001**	**<0.0001**	
DM	Average	40,329	4,851	
	SD	36,680	4,096	
	Total number	128	128	
	Positive number	17	13	
	Positive (%)	**13.3%**	**10.2%**	
	*P* value (*vs* HD)	**<0.0001**	**<0.0001**	

The upper panel indicates the number of total samples, samples from male and female participants, and ages (average ± SD). The lower panel summarizes the s-CSF2-Ab and s-CSF2pep-Ab levels examined using AlphaLISA. Numbers are as shown in Table 1; *P* values of <0.05 and positive rates of >10% are marked in bold font. The plots for these data are shown in Figures 3A,B. AMI, acute myocardial infarction; DM, diabetes mellitus.

The patients with DM, who were followed up for 100 months after serum collection, were categorized into positive and negative groups based on the cutoff s-CSF2-Ab and s-CSF2pep-Ab levels determined using the ROC analysis (Figures 3E,G). Albeit not correlated with age, sex, obesity, and the onset of cerebrovascular events or cancer, the s-CSF2pep-Ab positivity was significantly correlated with the onset of cardiovascular events (Supplementary Table S1). Furthermore, both the s-CSF2-Ab-and s-CSF2pep-Ab-positive groups had poor DM prognosis compared with the antibody-negative groups (*P* = 0.019 and *P* = 0.034, respectively; Figures 3C,H). Importantly, the difference in the overall survival between the antibody-positive and antibody-negative groups was most striking after 80 months.

### 3.4. Association of the s-CSF2-Ab and s-CSF2pep-Ab levels with CKD

**Figure 4:**
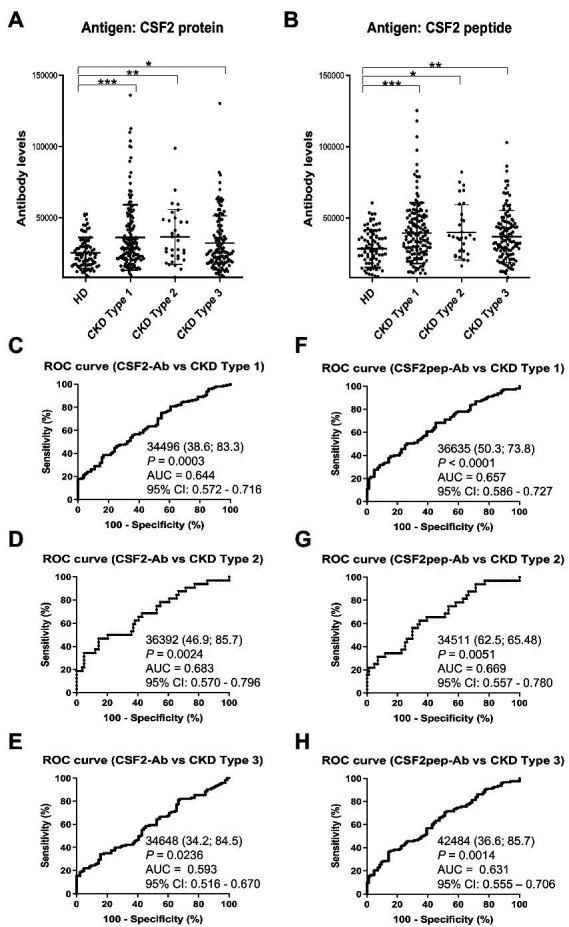
Association of the s-CSF2-Ab and s-CSF2pep-Ab levels with CKD. The levels of s-CSF-Ab (**A**) and s-CSF2pep-Ab (**B**) in HDs and in patients with diabetic CKD (CKD type 1), nephrosclerosis (CKD type 2), and glomerulonephritis (CKD type 3) were examined using AlphaLISA. The bars represent averages ± SD. *P < 0.05; **P < 0.01; ***P < 0.001, Kruskal–Wallis test. The data are summarized in Table 3. (**C–E**) ROC analysis to determine the ability of s-CSF2-Ab to predict CKD type 1 (**C**), type 2 (**D**), and type 3 (**E**). (**F–H**) ROC analysis to determine the ability of s-CSF2pep-Ab to predict CKD type 1 (**F**), type 2 (**G**), and type 3 (**H**). The numbers in the graphs are the same as those presented Figure 2. CKD, chronic kidney disease.

We also examined the s-CSF2-Ab and s-CSF2pep-Ab levels in the sera of 82 HDs and 300 patients with CKD, including 145 patients with diabetic kidney disease (CKD type 1), 32 patients with nephrosclerosis (CKD type 2), and 123 patients with glomerulonephritis (CKD type 3). The s-CSF2-Ab and s-CSF2pep-Ab levels were significantly higher in patients with CKD types 1, 2, and 3 than in HDs (Figures 4A,B). Based on the cutoff antibody levels, defined as two SDs above the average levels in HDs, the s-CSF2-Ab positivity rates were 4.8, 22.1, 28.1, and 22%, and the s-CSF2pep-Ab positivity rates were 1.2, 18.6, 21.9, and 14.6% in HDs and patients with CKD types 1, 2, and 3, respectively (Table 3). The ROC analysis revealed that the AUCs for s-CSF2-Ab were 0.644, 0.683, and 0.593 (Figures 4C–E), and the AUCs for s-CSF2pep-Ab were 0.657, 0.669, and 0.631 (Figures 4F–H) in patients with CKD types 1, 2, and 3, respectively. The AUC for the s-CSF2-Ab level was higher in patients with CKD type 2 than in those with CKD type 1.

**Table 3:** The s-CSF2-Ab and s-CSF2pep levels associated with CKD.

Sample information		HD	CKD type 1	CKD type 2	CKD type 3
Total sample number		82	145	32	123
Male/Female		44/38	106/39	21/11	70/53	Age (Average ± SD)	44.10 ± 11.19	66.04 ± 10.38	76.03 ± 9.78	61.98 ± 11.69
				
		s-CSF2-Ab	s-CSFpep-Ab		
HD	Average	25,266	28,353		
	SD	10,846	13,215		
	Cutoff value	46,958	54,783		
	Total number	84	84		
	Positive number	4	1		
	Positive (%)	4.8%	1.2%		
CKD type 1	Average	36,041	39,262		
	SD	22,993	21,382		
	Total number	145	145		
	Positive number	32	27		
	Positive (%)	**22.1%**	**18.6%**		
	*P* value (*vs* HD)	**<0.001**	**<0.001**		
CKD type 2	Average	36,425	39,778		
	SD	19,287	19,499		
	Total number	32	32		
	Positive number	9	7		
	Positive (%)	**28.1%**	**21.9%**		
	*P* value (*vs* HD)	**<0.01**	**<0.05**		
CKD type 3	Average	32,164	36,810		
	SD	19,073	18,290		
	Total number	123	123		
	Positive number	27	18		
	Positive (%)	**22.0%**	**14.6%**		
	*P* value (*vs* HD)	**<0.05**	**<0.01**		

The upper panel indicates the number of total samples, samples from male and female participants, and ages (average ± SD). The lower panel summarizes the s-CSF2-Ab and s-CSF2pep-Ab levels examined using AlphaLISA. Numbers are as shown in Table 1; *P* values of <0.05 and positive rates of >10% are marked in bold font. The plots for these data are shown in Figures 4A,B. CKD, chronic kidney disease; CKD type 1, diabetic CKD; CKD type 2, nephrosclerosis; CKD type-3, glomerulonephritis.

### 3.5. Correlation analysis

We conducted a comparative analysis of the s-CSF2-Ab levels and participant characteristics in a cohort of 384 participants from Chiba Prefectural Sawara Hospital, including 196 patients with AIS, 79 patients with TIA, and 109 HDs. The baseline characteristics of the cohort are summarized in Supplementary Table S2. The Mann–Whitney *U* test was employed to compare the s-CSF2-Ab levels between males and females; obese or not (body mass index, < or ≥ 25 kg/m^2^); those with (+) or without (−) another disease including DM, hypertension, CVD, and dyslipidemia; and those with or without smoking and alcohol intake habits. Significantly higher s-CSF2-Ab levels were observed in patients with hypertension than in those without hypertension (*P* = 0.020; Table 4). Contrarily, the s-CSF2-Ab levels were inversely associated with dyslipidemia (*P* = 0.027) possibly because the patients but not the HDs were taking cholesterol-lowering drugs, such as statins. However, s-CSF2-Ab showed lower levels in participants with CVD, probably because only 15 positive cases with CVD complications were compared with 369 negative cases with CVD complications (*P* = 0.976).

**Table 4:** Association between s-CSF2-Ab levels and the data from participants in the Sawara Hospital cohort.

Data analysis of s-CSF2-Ab levels
Sex		Male	Female	Sample number		230	154
s-CSF2-Ab level	Average	7,553	6,374
	SD	6,771	5,083
*P* value (*vs* Male)			0.166
Obesity		BMI < 25	BMI ≥25
Sample number		263	121
s-CSF2-Ab level	Average	7,288	6,628
	SD	6,622	5,046
*P* value (*vs* BMI < 25)			0.270
Complication		DM-	DM+
Sample number		303	81
s-CSF2-Ab level	Average	7,062	7,150
	SD	5,900	7,129
*P* value (*vs* DM-)			0.909
Complication		Hypertension-	Hypertension+
Sample number		144	240
s-CSF2-Ab level	Average	6,105	7,666
	SD	4,927	6,750
*P* value (*vs* Hypertension-)			**0.020**
Complication		CVD-	CVD+
Sample number		369	15
s-CSF2-Ab level	Average	7,124	5,999
	SD	6,271	2,498
*P* value (*vs* CVD-)			0.976
Complication		Dyslipidemia-	Dyslipidemia+
Sample number		267	117
s-CSF2-Ab level	Average	7,608	5,876
	SD	6,862	3,950
*P* value (*vs* Dyslipidemia-)			**0.027**
Lifestyle		Non-smoker	Smoker
Sample number		187	197
s-CSF2-Ab level	Average	6,705	7,493
	SD	6,580	5,675
*P* value (*vs* Non-smoker)			0.091
Lifestyle		Alcohol-	Alcohol+
Sample number		173	211
s-CSF2-Ab level	Average	7,258	6,935
	SD	5,486	6,688
*P* value (*vs* Alcohol-)			0.128

Participants were divided into two groups according to the following classifications: sex (male and female); obesity (body mass index, < or ≥ 25 kg/m^2^); with (+) or without (−) other disease including DM, hypertension, CVD, and dyslipidemia; and with (+) or without (−) smoking and alcohol intake habits. The Mann–Whitney *U* test was used to compare the s-CSF2-Ab levels of the two groups. Sample numbers, averages, and SDs of counts, as well as *P* values are shown. Significant correlations (*P* < 0.05) are marked in bold.

Furthermore, Spearman’s rank-order correlation analysis was conducted to determine the correlation between the serum antibody levels and participant characteristics, including general information (e.g., age, body height, weight, body mass index, and degree of artery stenosis) and blood tests (sodium, chloride, white and red blood cell counts, hematocrit, etc.). The s-CSF2-Ab levels were significantly correlated with age, blood pressure (BP), smoking duration, and max-IMT (Table 5). However, the s-CSF2-Ab levels were inversely correlated with the levels of total cholesterol, cholinesterase, and albumin. The observed correlation with max-IMT suggested that s-CSF2-Ab levels were associated with early atherosclerosis prior to stenosis (62–64).

**Table 5:** Correlation analysis of s-CSF2-Ab levels with data from subjects in the Sawara Hospital cohort.

	s-CSF2-Ab	
Category	*r* value	*P* value
Age	0.0969	**0.0381**
Height (cm)	0.0478	0.3522
Weight (kg)	−0.0502	0.3276
BMI	−0.0961	0.0609
max IMT	0.1579	**0.0056**
A/G	−0.0964	0.0681
AST (GOT)	−0.0576	0.2620
ALT (GPT)	−0.0732	0.1535
ALP	0.0522	0.3302
LDH	0.0395	0.4483
tBil	−0.0543	0.2955
CHE	−0.1560	**0.0081**
gamma-GTP	−0.0916	0.0822
albumin	−0.1140	**0.0263**
BUN	0.0490	0.3391
creatinin	0.0770	0.1332
eGFR	−0.0583	0.2700
UA	0.0628	0.3008
AMY	−0.0735	0.2724
T-CHO	−0.1889	**0.0006**
HDL-c	−0.0512	0.4406
TG	−0.0315	0.6219
Na	−0.0025	0.9608
Cl	−0.0477	0.3554
WBC	0.0575	0.2633
RBC	0.0370	0.4726
HGB	−0.1067	**0.0377**
HCT	−0.1096	**0.0326**
MCV	−0.0581	0.2584
MCH	−0.0023	0.9636
MCHC	−0.0583	0.2572
PLT	−0.0071	0.8896
MPV	0.0222	0.6658
PCT	0.0414	0.4303
PDW	−0.0129	0.8027
Blood glucose	0.0995	0.0632
HbA1c	−0.0444	0.4478
BP	0.1069	**0.0439**
Smoking duration (year)	0.1139	**0.0288**
Alcohol Freq (time/w)	0.0791	0.1297

Correlation coefficients (*r* values), and *P* values obtained by Spearman correlation analysis are shown. Subjects’ data used were age, height, weight, BMI, body mass index; max-IMT, maximum intima–media thickness; A/G, albumin/globulin ratio; AST, aspartate aminotransferase; ALT, alanine amino transferase; ALP, alkaline phosphatase; LDH, lactate dehydrogenase; tBil, total bilirubin; CHE, cholinesterase; gamma-GTP, γ-glutamyl transpeptidase; ALB, albumin; BUN, blood urea nitrogen; creatinine; eGFR, estimated glomerular filtration rate; UA, uric acid; AMY, amylase; T-CHO, total cholesterol; HDL-c, high-density lipoprotein cholesterol; TG, triglyceride; Na, sodium; Cl, chloride; WBC, white blood cell number; RBC, red blood cell number; HGB, hemoglobin; HCT, hematocrit; MCV, mean corpuscular volume; MCH, mean corpuscular hemoglobin; MCHC, MCH concentration; PLT, platelet number; MPV, mean platelet volume; PCT, procalcitonin; PDW, platelet distribution width; HbA1c, blood glucose, glycated hemoglobin; smoking duration (year), and alcohol intake frequency (times/week).

Spearman’s rank-order correlation analysis of the CKD cohort consisting of 300 participants also revealed a significant correlation with plaque score, max-IMT, and cardio-ankle vascular index (left and right; Supplementary Table S3), which reflect the degree of atherosclerosis (65). Furthermore, C-reactive protein was associated with the s-CSF2-Ab levels in the CKD cohort, supporting the role of s-CSF2-Ab in inflammation in CKD.

Logistic regression analysis was conducted to determine the predictors of AIS in the Chiba Prefectural Sawara Hospital cohort (Supplementary Table S2). The univariate logistic regression analysis revealed that elevated s-CSF2-Ab levels were associated with increased risk of AIS (*P* < 0.0001). Moreover, the multivariate logistic regression analysis demonstrated that age, hypertension, and s-CSF2-Ab level were independent predictors of AIS (Supplementary Table S4).

### 3.6. Japan public health center cohort analysis

To determine whether s-CSF2pep-Ab can be used as a marker to predict the onset of AIS, we examined the JPHC cohort samples, which were obtained as previously described (30). The s-CSF2pep-Ab levels were strongly associated with the risk of AIS. The ORs (95% CI) were 1.72 (0.91–3.24), 2.47 (1.31–4.65), and 3.03 (1.58–5.80) for the samples with the second, third, and fourth quartiles of the s-CSF2pep-Ab levels, respectively, compared with the first quartile (Table 6). Consistently, the s-CSF2pep-Ab levels were significantly higher in patients with TIA (Figure 2E), which can be a prodromal stage of AIS (66), compared with HDs. These results indicated that s-CSF2pep-Ab might be a useful marker in predicting the onset of AIS.

**Table 6:** Analysis of JPHC cohort subjects.

s-CSF2pep-Ab *vs* AIS	Case/control	Matched OR (95% CI)
1st	28/51	1.00
2nd	40/50	1.72 (0.91–3.24)
3rd	61/51	2.47 (1.31–4.65)
4th	73/50	3.03 (1.58–5.80)

The odds ratios (ORs) and 95% CI of the 1st, 2nd, 3rd, and the highest (4th) quartiles versus the lowest (1st) quartile are shown for AIS with respect to anti-CSF2 peptide antibody (CSF2pep-Ab) levels. Age-, sex-, and area-matched, conditional odds ratios, and 95% confidence intervals (CIs) of AIS according to CSF2 peptide antibody markers. OR: odds ratios.

### 3.7. Elevated s-CSF2-Ab levels in patients with cancer

**Figure 5:**
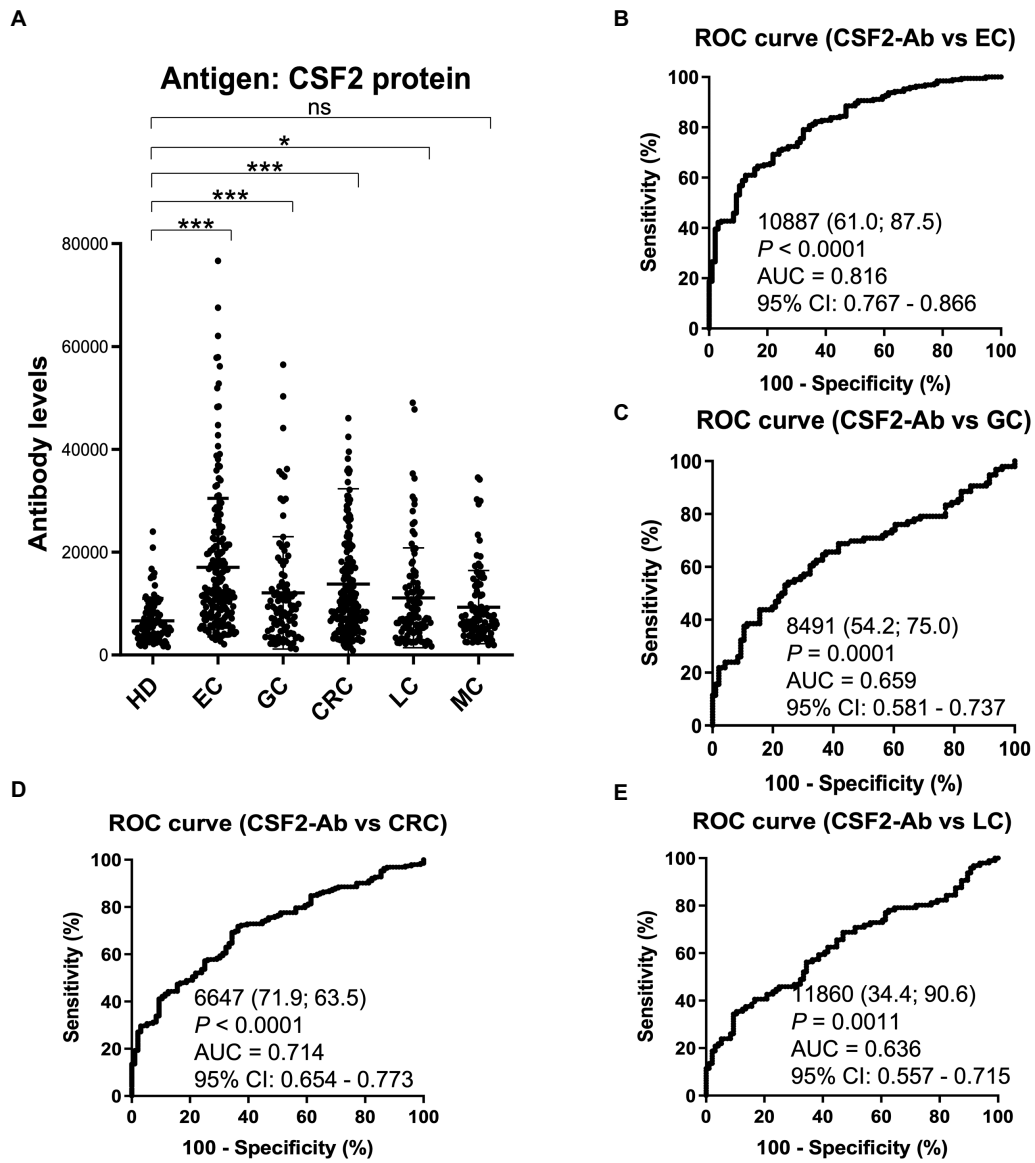
Comparison of the s-CSF2-Ab levels between HDs and patients with cancers. (**A**) Scatter dot plots of the s-CSF2-Ab levels in HDs and in patients with EC, gastric cancer (GC), CRC, lung cancer (LC), and mammary cancer (MC). The results are presented as described in the legend of Figure 2. **P < 0.01; ***P < 0.001; ns, not significant. ROC analysis was conducted to assess the ability of s-CSF2-Abs to detect EC (**B**), GC (**C**), CRC (**D**), and LC (**E**).

Based on previous reports suggesting that CSF2 could inhibit the development of cancer (13–15, 67), we further investigated the serum samples from patients with EC, CRC, GC, LC, and MC. The s-CSF2-Ab levels were significantly higher in patients with EC, CRC, GC, and LC, but not in those with MC, compared with HDs (Figure 5; Table 7). The highest rates of s-CSF2-Ab positivity were observed in patients with EC and CRC (Table 7). The AUCs for s-CSF2-Ab were 0.816 and 0.714 versus EC and CRC, respectively (Figures 5B,D), which were much higher than versus AIS (0.576), AMI (0.650), DM (0.638), type-1 CKD (0.644), type-2 CKD (0.683), type-3 CKD (0.593), GC (0.659), and LC (0.636; Figures 2C, 3D,E, 4C–E, 5B–E). This may indicate that s-CSF2-Ab levels are more closely associated with EC and CRC compared to others.

**Table 7:** Association of s-CSF2-Ab levels with cancer development.

Sample information		HD	EC	GC	CC	LC	MC
Total sample number		96	192	96	192	96	96
Male/Female		51/45	155/37	68/28	119/74	42/54	58/38
Age (Average ± SD)		57.9 ± 6.0	67.4 ± 9.8	68.7 ± 10.6	66.7 ± 11.7	60.9 ± 13.3	68.1 ± 9.6			
		s-CSF2-Ab					
HD	Average	6,678					
	SD	4,312					
	Cutoff value	15,301					
	Total number	96					
	Positive number	5					
	Positive (%)	5.2%					
EC	Average	17,068					
	SD	13,393					
	Total number	192					
	Positive number	82					
	Positive (%)	**42.7%**					
	*P* value (*vs* HD)	**<0.0001**					
GC	Average	12,095					
	SD	10,906					
	Total number	96					
	Positive number	23					
	Positive (%)	**24.0%**					
	*P* value (*vs* HD)	**<0.0001**					
CRC	Average	13,779					
	SD	18,560					
	Total number	192					
	Positive number	58					
	Positive (%)	**30.2%**					
	*P* value (*vs* HD)	**<0.0001**					
LC	Average	11,127					
	SD	9,695					
	Total number	96					
	Positive number	23					
	Positive (%)	**24.0%**					
	*P* value (*vs* HD)	**<0.05**					
MC	Average	9,320					
	SD	7,120					
	Total number	96					
	Positive number	15					
	Positive (%)	**15.6%**					
	*P* value (*vs* HD)	ns					

Types of cancer diagnoses included. EC, esophageal cancer; GC, gastric cancer; CRC, colorectal carcinoma; LC, lung cancer; MC, mammary cancer. The upper panel indicates the numbers of all samples and samples from males and females as well as age (average ± SD). The lower panel summarizes the serum antibody levels examined by AlphaLISA using purified CSF2-GST proteins as the antigens, as described in the legend of Table 1. *P* values of < 0.05 and positive rates of > 10% are marked in bold font. The plots for these data are shown in Figure 5A.

**Figure 6:**
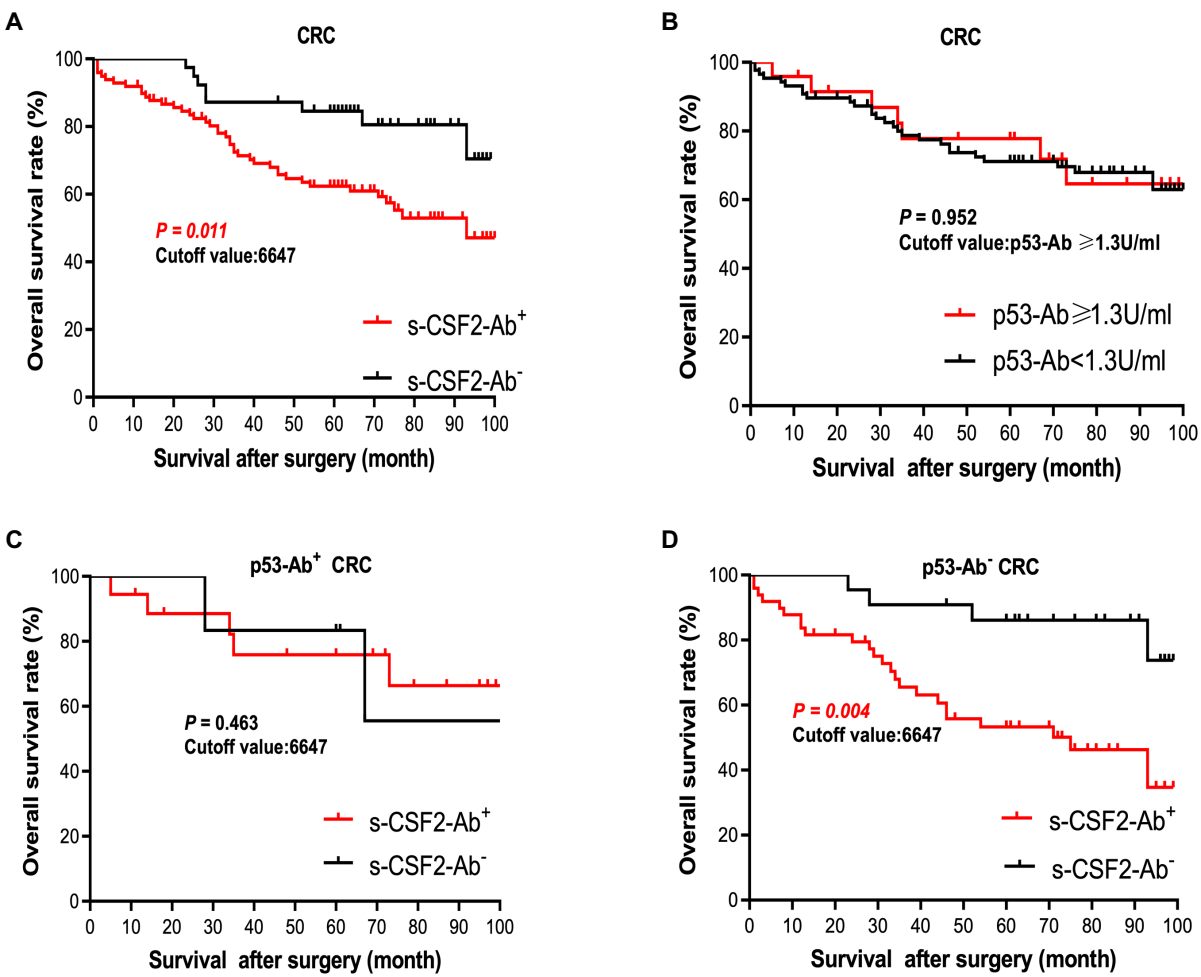
Comparison of survival according to s-CSF2-Ab positivity in patients with CRC after surgery. (**A**) Comparison of the postoperative survival between the s-CSF2-Ab^+^ and s-CSF2-Ab^−^ patients with CRC (*P* = 0.011). (**B**) Comparison of the overall survival between the p53-Ab^+^ (≥1.3 U/ml) and p53-Ab^−^ (<1.3 U/ml) patients with CRC (*P* = 0.952). (**C**) Comparison of the overall survival between the s-CSF2-Ab^+^ and s-CSF2-Ab^−^ patients with CRC among those with p53-Ab ≥1.3 U/ml (*P* = 0.463). (**D**) Comparison of the overall survival between the s-CSF2-Ab^+^ and s-CSF2-Ab^−^ patients with CRC among those with p53-Ab <1.3 U/ml (*P* = 0.004). The cutoff values in patients with CRC were determined *via* the ROC analysis presented in Figure 5D. Statistical analyzes were performed by the Log-Rank test between two groups.

The patients with CRC were divided into the positive and negative groups based on the cutoff s-CSF2-Ab levels determined using the ROC analysis (Figure 5D) to evaluate the association of the s-CSF2-Ab levels with postoperative survival and all of whom underwent radical surgery. The overall survival was significantly different between the s-CSF2-Ab-positive and s-CSF2-Ab-negative patients with CRC (*P* = 0.011; Figure 6A). Moreover, we analyzed the correlation between s-CSF2-Ab and the age or the stage in patients with CRC. The result revealed that the s-CSF2-Ab was independent of the age or the stage (Supplementary Table S5). The comparison of the overall survival in patients with CRC categorized according to serum p53-Ab positivity (i.e., serum p53-Ab ≥1.3 IU/ml) did not reveal a significant difference (*P* = 0.952; Figure 6B).

The correlation analysis of patients with CRC indicated that the s-CSF2-Ab levels were associated with the serum p53-Ab level (*P* = 0.039) but not with the serum CEA or CA19-9 levels (Table 8), suggesting a degree of functional relevance between the serum CSF2 and p53 levels. Next, we categorized the patients with CRC into p53-Ab-positive and p53-Ab-negative populations. Within the p53-Ab-negative group, the prognosis of the s-CSF2-Ab-positive patients was significantly worse than that of the s-CSF2-Ab-negative patients (*P* = 0.004), which was not observed within the p53-Ab-positive group (Figures 6C,D). Serum p53-Ab-negative cancers may be maintaining functional wild-type p53; therefore, these results raised the possibility that s-CSF2-Ab and CSF2 could influence the development of CRC *via* wild-type p53. Of note, the overall survival based on the s-CSF2-Ab levels did not exhibit significant differences in patients with EC and GC (Supplementary Figures 3A,B).

**Table 8:** Association between s-CSF2-Ab levels and data from participants in the Toho University Hospital cohort.

Data analysis of s-CSF2-Ab levels
Sex		Male	Female	Sample number		45	68
s-CSF2-Ab level	Average	10,178	6,374
	SD	7,451	5,083
*P* value (*vs* Male)			**0.010**
CEA		CEA ≥10 ng/ml	CEA <10 ng/ml
Sample number		29	84
s-CSF2-Ab level	Average	14,744	12,333
	SD	11,892	8,825
*P* value (*vs* CEA < 10 ng/ml)			0.428
CA19-9		CA19-9 ≥ 37 ng/ml	CA19-9 < 37 ng/ml
Sample number		58	55
s-CSF2-Ab level	Average	13,525	12,347
	SD	10,331	9,052
*P* value (*vs* CA19-9 < 37 ng/ml)			0.436
p53-Abs		p53-Ab ≥1.3 U/ml	p53-Ab <1.3 U/ml
Sample number		24	89
s-CSF2-Ab level	Average	17,158	11,818
	SD	11,977	8,732
*P* value (*vs* p53-Ab<1.3 U/ml)			**0.039**
Organizational type		Tubular	None-Tubular
Sample number		50	63
s-CSF2-Ab level	Average	13,355	12,632
	SD	10,182	9,379
*P* value (*vs* none-tubular)			0.555

Participants were divided into two groups according to the following parameters: sex (male and female), obesity, BMI, body mass index; CEA, carcinoembryonic antigen; CA19-9, carbohydrate antigen 19–9; p53-Ab, and organizational type. The Mann–Whitney *U* test was used to compare the s-CSF2-Ab levels of the two groups. Sample numbers, averages, and SDs of counts, as well as *P* values are shown. Significant correlations (*P* < 0.05) are marked in bold.

### 3.8. Effect of CSF2 and anti-CSF2 antibodies on cell replication and transactivation ability of p53 in CRC cells

We evaluated the effects of the CSF2 and anti-CSF2 antibodies on cell replication using LoVo cells, a human CRC line harboring wild-type p53 (55). LoVo cells were treated with CSF2 and/or a commercial rabbit polyclonal anti-CSF2 antibody (Cusabio Technology) to examine their impact on cell replication using the MTS assay. As presented in Supplementary Figure S4, treatment with CSF2 for 72 h led to a slight decrease in cell replication, whereas the presence of the anti-CSF2 antibody significantly promoted the proliferation of LoVo cells. Moreover, the replication-promoting effect of the anti-CSF2 antibody was reversed by the simultaneous addition of an excess quantity of antigenic CSF2 (PeproTech), suggesting that the stimulation of proliferation by the anti-CSF2 antibody was attributed mainly to the suppression of CSF2 in the medium and not to other effects due to impurities. Contrarily, none of the effects of CSF2 and the anti-CSF2 antibody observed in LoVo cells were detected in DLD1 harboring mutant p53 (Supplementary Figure S4).

Next, we used pGL3-p21-Luc containing the promoter sequence of *p21/Cip1*, a p53-target gene, to evaluate the p53 activity after the addition of CSF2 and/or the anti-CSF2 antibody using a luciferase reporter assay. In LoVo cells, p21-Luc, which was partially preactivated by cotransfection of wild-type p53, was further activated by treatment with CSF2 but significantly attenuated by treatment with the anti-CSF2 antibody (*P* < 0.01; Supplementary Figure S5). The decrease in the activation of p21-Luc by the anti-CSF2 antibody was reversed by the concurrent addition of excess antigenic CSF2, indicating that the reduction of p21-Luc activation by the anti-CSF2 antibody was caused by the suppression of CSF2 in the medium rather than its effects related to other impurities in the CSF2 antibody preparation. Therefore, these results indicate that CSF2 might induce the p53 transactivation ability, leading to the inhibition of tumor cell proliferation.

To determine p53-mediated transactivation, we measured the phosphorylation of p53 at Ser-46 using western blotting (68). The levels of Ser-46-phosphorylated p53 were reduced after treatment with the rabbit anti-CSF2 antibody and increased after treatment with CSF2 (Supplementary Figure S6). No apparent changes were observed in the levels of p53 phosphorylated at other sites, including Ser-392, Ser-315, and Thr-81 (data not shown). Thus, these results indicated that the rabbit anti-CSF2 antibody and the serum autoantibodies against CSF2 promoted tumor progression by suppressing p53 activity, which was preactivated by endogenous CSF2.

## 4. Discussion

In the present study, we investigated the relationship between CSF2 and atherosclerosis-related diseases, including AIS, AMI, DM, CKD, and cancer based on data from previous studies. We confirmed the presence of anti-CSF antibodies in patient sera using western blotting with recombinant full-length GST-tagged CSF2 (Figure 1). Subsequently, we measured the serum antibody levels using GST-CSF2 and the *N*-terminal biotinylated 18-mer peptide, bCSF2-77, as antigens in AlphaLISA. We found that both the s-CSF2-Ab and s-CSF2pep-Ab levels were higher in patients with AIS, AMI, DM, and CKD than those with HDs (Figures 2, 3, 5; Tables 1–3). The s-CSF2-Ab levels were closely correlated with max-IMT, plaque score, and cardio-ankle vascular index (Table 5; Supplementary Table S3), which are typical indices of atherosclerosis leading to AIS and AMI (62–65). CSF2 has been demonstrated to have a protective impact on atherogenesis (6), and the administration of CSF2 has been reported to prevent the progression of atherosclerosis *via* changes in the composition of atherosclerotic lesions (7). We observed that both the s-CSF2-Ab and s-CSF2pep-Ab levels were highly correlated with atherosclerosis-related diseases, such as AIS, AMI, CKD, and DM. Intriguingly, we also found that the levels of s-CSF2pep-Ab but not s-CSF2-Ab were associated with TIA. It is possible that the CSF2 peptide with a single epitope can be specifically recognized by anti-CSF2 antibodies, whereas the CSF2 protein with multiple epitopes can also cross-react with other non-specific antibodies in addition to being recognized by specific anti-CSF2 antibodies. Furthermore, we found that the AUC of s-CSF2pep-Ab in patients with AIS, AMI, DM, and CKD were all higher than the AUC of s-CSF2-Ab in patients, except for type-2 CKD. Consistent with this, it was reported that using peptide epitopes would be beneficial with respect to assay specificity, while recombinant proteins also included many cross-reactive epitopes that would react with low specificity antibodies, leading to a lower specificity of the test (48, 69). Therefore, the use of s-CSF2pep-Ab as an antibody marker may be more accurate for the diagnosis of diseases. The s-CSF2-Ab levels were correlated well with hypertension (Table 4) and weakly with BP (Table 5) but were not correlated with glycated hemoglobin and blood glucose, which are typical DM markers. Patients with diabetic CKD (CKD type 1) and those with nephrosclerosis (CKD type 2) exhibited equally higher s-CSF2-Ab and s-CSF2pep-Ab levels than the HDs (Figures 4A, B). The AUCs of CKD type 2 were higher than those of CKD type 1. These results indicate that the s-CSF2-Ab levels do not directly reflect DM but are associated with DM-induced atherosclerotic disorders. Furthermore, using multivariate logistic regression analysis, we identified age, hypertension, and s-CSF2-Ab as independent predictors of AIS (Supplementary Table S4). Thus, s-CSF2-Ab may be able to discriminate atherosclerosis caused by hypertension, which leads to the onset of AIS and AMI. These findings, subsequently validated by the analysis of the JPHC cohort (Table 6), indicate that s-CSF2-Ab should be considered as a risk factor for AIS.

In addition, the s-CSF2-Ab levels were associated with several cancers, including EC, CRC, GC, and LC (Figure 5; Table 7). As presented in Table 8, the s-CSF2-Ab and p53-Ab levels were correlated. Among the patients with CRC whose serum p53-Ab levels were below the cutoff, the prognosis was worse in those with high s-CSF2-Ab levels than in those with low s-CSF2-Ab levels. Furthermore, the *P* value of Figure 6D was even lower than that of Figure 6A in the survival analysis of patients with CRC. After transactivation by the wild-type p53, the protein product of mouse double minute 2 homolog (*MDM2*) preferentially polyubiquitinates p53 protein, leading to its degradation (68, 70). Contrarily, in the presence of mutant p53, the MDM2 levels are maintained at low levels, and the degradation of p53 is limited, leading to the high p53 expression (71). Therefore, most of the anti-p53 autoantibodies in patients with CRC are formed in response to p53 mutations (72), and patients without p53-Ab harbor wild-type p53. Based on the observed effect of p53-Ab status, we used wild-type p53-harboring LoVo cells to investigate the relationship of CSF2 and CSF2-Ab with p53 (Supplementary Figures S4, S5). We found that anti-CSF2-Ab reduced the p53 transactivation ability, leading to the increased proliferation of LoVo cells (Supplementary Figures S4, S5). Consistently, the levels of Ser-46-phosphorylated p53 were increased by treatment with CSF2 and reduced by treatment with anti-CSF2-Ab (Supplementary Figure S6). Interestingly, it was previously reported that the anti-CSF2 antibodies decreased CSF2 cytokine bioavailability, possibly by disrupting their binding to receptors (73–75), and the expression of p53 was significantly increased in neutrophils cultured with CSF2 (76). These results indicate that the high levels of CSF2-Ab may reduce p53 activity *via* adsorption of CSF2 in the circulation, leading to an increase in the proliferation of cancer cells and unfavorable prognosis in patients with CRC (Figure 6D). Gene therapy using wild-type p53, delivered using physical methods or viral vectors, was reported to significantly suppress CRC (77), but not MC (78), in preclinical and clinical models. Therefore, CRC might be more sensitive to the tumor suppressor p53 than MC, which might partially explain our findings that the s-CSF2-Ab levels were not associated with MC (Figure 5; Table 7).

**Figure 7:**
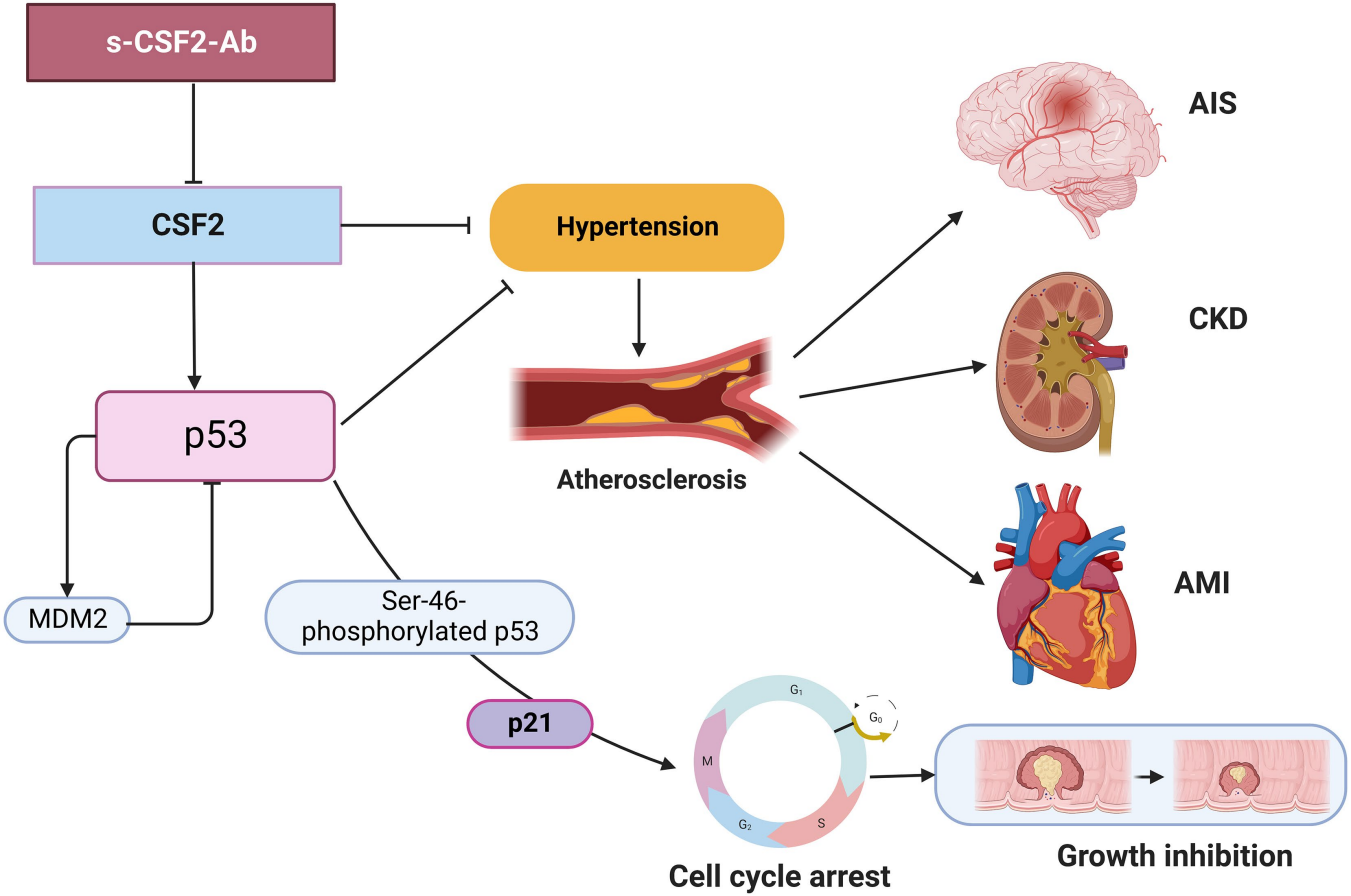
Diagram of the relationship between s-CSF2-Ab and p53 in cancer and atherosclerosis-related diseases. Arrows represent the downstream activation, and blocked arrows represent inhibitory action. Created with BioRender.com. *MDM2*, mouse double minute 2 homolog.

Numerous lines of evidence support the presence of a relationship between atherosclerosis and cancer, which share inflammation as an underlying pathophysiology (11, 12). p53 deficiency not only leads to accelerated atherosclerosis (79–81) but also increases BP, heart rate, and sympathetic activity of the heart and blood vessels, thus contributing to the development of hypertension (82). In the present study, we found that the s-CSF2-Ab levels were significantly associated with hypertension, which is a major risk factor for not only atherosclerosis (9, 83–85) but also AIS and AMI (35, 86–88). Therefore, based on the current study findings, we hypothesize that the s-CSF2-Ab levels may contribute to atherosclerosis through the elevated hypertension caused by reduced p53 activity. Therefore, based on the current study findings, we hypothesize that the s-CSF2-Ab may directly contribute to the development of cancer by reducing p53 activity, and indirectly contribute to the onset of atherosclerosis-related AIS, AMI, and CKD through p53-mediated regulation of hypertension. This may explain the overall higher positive rate of s-CSF2-Ab in patients with solid cancer than those in patients with atherosclerosis-related AIS, AMI, DM, and CKD (Tables 1–3, 7). The overview diagram (Figure 7) summarizes the relationship between s-CSF2-Ab and wild-type p53 in cancer and atherosclerosis-related diseases. Consistently, the high s-CSF2-Ab levels in patients with DM indicated unfavorable prognosis, which was especially pronounced after 80 months (Figures 3C,H). Thus, s-CSF2-Ab might have a causal relationship with the development of atherosclerosis and cancer.

Results of this study suggest that the measurement of serum s-CSF2-Ab levels can provide valuable information for diagnosing atherosclerosis-related AIS and solid cancers. The serum s-CSF2pep-Ab levels can be used for diagnosing TIA. Investigations showed that approximately 15% of patients with ischemic stroke experience a TIA before the onset of AIS (89), and approximately 50% of the patients with TIA visited medical facilities after their symptoms completely disappeared (90, 91). However, it is sometimes difficult for physicians to diagnose a TIA only by taking the patient’s history into consideration, as half of the TIA patients visit the hospital after the symptoms have completely disappeared. Although tests such as MRI and carotid artery angiography can be used to improve diagnoses, they are generally expensive, time consuming, and inconvenient. In the case of solid cancers, early diagnoses can greatly improve the survival time of patients and reduce the financial burden caused by medical treatments. Therefore, if s-CSF2pep-Ab can be applied as a biomarker in combination with other risk factors and biomarkers for AIS, TIA or solid cancers to diagnose the occurrence of the disease, this will greatly facilitate clinical practice and contribute to the medical economy. Cytokines and cytokine antibodies in the serum are the ideal targets for the development of preventative and therapeutic approaches due to drug accessibility. The administration of CSF2 has been demonstrated to prevent the progression of atherosclerosis (7). Alternatively, the development decoy molecules that can bind to and adsorb s-CSF2-Ab might be effective for disease prevention.

## 5. Limitation

We acknowledge that the current study has several limitations. First, atherosclerosis risk factors, such as hypercholesterolemia, were not associated with the elevated levels of s-CSF2-Ab in the present study. Antihypertensive drugs, cholesterol-lowering statins, and antiplatelet agents are considered to influence the pathogenesis of atherosclerosis (92–95). Because detailed statistics on medications taken by patients prior to hospital admission are not available, we were unable to study the specific effects of drugs on s-CSF2-Ab levels, and further studies are needed in patients who were not taking drugs that could affect atherosclerosis and cancer. In future studies, the potential modulating effects of these drugs on the s-CSF2-Ab levels should be considered. Second, the blood samples were collected at the time of hospital admission and the period of stroke onset and admission differed depending on the patient, which is another limitation. Third, as we used specimens obtained from hospitals and universities in Japan, it is unclear whether our conclusions are applicable to other populations. Thus, it is essential for general practical use in the world to conduct more international collaborative research using specimens from many countries. Fourth, the cutoff values we used were average+2SD and Youden index of ROC analysis. However, other methods should also be considered to optimize the cutoff values, improving the ability of s-CSF2-Ab as a biomarker to discriminate normal individuals from patients and favorable prognosis from unfavorable prognosis.

## 6. Conclusion

It is possible to predict the onset of atherosclerotic AIS and AMI by using s-CSF2-Ab as a marker. Moreover, high s-CSF2-Ab levels exhibited poor overall survival in patients with CRC who may be harboring wild-type p53. Taken together, these results indicate s-CSF2-Ab as a potentially valuable therapeutic target for the prevention of atherosclerosis and solid cancers.

## Data availability statement

The original contributions presented in the study are included in the article/Supplementary material, further inquiries can be directed to the corresponding authors.

## Ethics statement

The studies involving human participants were reviewed and approved by Local Ethics Review Board of Chiba University, Graduate School of Medicine in Chiba, Japan (no. 2018–320), Toho University Graduate School of Medicine in Tokyo, Japan (no. A19033). The patients/participants provided their written informed consent to participate in this study.

## Author contributions

SYL, TMac, YI, and TH conceived and designed the study. SYL, MK, TMac, KYa, MO, MT, and KI conducted the experiments and acquired the data. YY, SM, MI, KYos, AH, KYok, HI, KK, NS, ST, and HT contributed to the reagents, materials, analysis tools, or the patient data. YY, SY, MS, EN, and IK analyzed and interpreted the data. BSZ, TMat, MI, HS, and MT performed the statistical analyzes. SYL, YI, and TH drafted the manuscript. All authors contributed to the article and approved the submitted version.

## Funding

This work was supported, in part, by research grants from the Japan Science and Technology Agency (JST), JSPS KAKENHI Grant Number 20 K17953, 19 K09451, 17 K19810, 20 K07810, 16 K10520, 21 K08695, and 15 K10117. The Japan Public Health Center-based Prospective Study was supported by (National Cancer Center Research and Development Fund since 2011) and a Grant-in-Aid for Cancer Research from the Ministry of Health, Labour and Welfare of Japan (from 1989 to 2010).

## Conflict of interest

The authors declare that the research was conducted in the absence of any commercial or financial relationships that could be construed as a potential conflict of interest.

## Publisher’s note

All claims expressed in this article are solely those of the authors and do not necessarily represent those of their affiliated organizations, or those of the publisher, the editors and the reviewers. Any product that may be evaluated in this article, or claim that may be made by its manufacturer, is not guaranteed or endorsed by the publisher.

## Footnotes

^1^ http://www.imtech.res.in/raghava/propred/
