# Supplementary material for: Utility of atherosclerosis-associated serum antibodies against colony-stimulating factor 2 in predicting the onset of acute ischemic stroke and prognosis of colorectal cancer
Source: Front Cardiovasc Med. 2023 Feb 10;10:1042272. doi: 10.3389/fcvm.2023.1042272 (PMC9954151; doi:10.3389/fcvm.2023.1042272)
Supplement: Supplementary file 1 [file Data_Sheet_1.docx]

Supplementary Material

**Utility of atherosclerosis-associated serum antibodies against colony-stimulating factor 2 in predicting the onset of acute ischemic stroke and prognosis of colorectal cancer**

Shu-Yang Li^1,2^*, Yoichi Yoshida^1,3^, Masaaki Kubota^1,2^, Bo-Shi Zhang^1^, Tomoo Matsutani^1^, Masaaki Ito^4^, Satoshi Yajima^5^, Kimihiko Yoshida^5^, Seiichiro Mine^1,6,7^, Toshio Machida^1,7,8^, Aiko Hayashi^9^, Minoru Takemoto^9,10^, Koutaro Yokote^9^, Mikiko Ohno^11,12^, Eiichiro Nishi^11,12^, Kenichiro Kitamura^13^, Ikuo Kamitsukasa^14^, Hirotaka Takizawa^15^, Mizuki Sata^16,17^, Kazumasa Yamagishi^16^, Hiroyasu Iso^18^, Norie Sawada^19^, Shoichiro Tsugane^19^, Katsuro Iwase^2^, Hideaki Shimada^4,5^, Yasuo Iwadate^1,3^, Takaki Hiwasa^1,2,3,4^*

***Co-corresponding authors:**

Takaki Hiwasa, Department of Neurological Surgery, Graduate School of Medicine, Chiba University, Inohana 1-8-1, Chuo-ku, Chiba 260-8670, Japan; e-mail: hiwasa_takaki@faculty.chiba-u.jp

Shu-Yang Li, Department of Neurological Surgery, Graduate School of Medicine, Chiba University, Inohana 1-8-1, Chuo-ku, Chiba 260-8670, Japan; e-mail: lishuyang1990@hotmail.com

# Supplementary Tables and Figures

**Supplementary Table 1.** Correlation analysis of patients with diabetes mellitus

| Age |  | ≥65 | <65 |
| --- | --- | --- | --- |
| Sample number | | 140 | 134 |
| s-CSF2-Ab level | Average | 4,032 | 4,010 |
|  | SD | 4,740 | 6,310 |
| *P* value (vs age<65) | |  | 0.110 |
|  |  |  |  |
| Sex |  | Male | Female |
| Sample number | | 119 | 155 |
| s-CSF2-Ab level | Average | 3,782 | 4,205 |
|  | SD | 4,064 | 6,476 |
| *P* value (vs Male) | |  | 0.836 |
|  |  |  |  |
| Obesity |  | BMI <25 | BMI ≥25 |
| Sample number | | 161 | 113 |
| s-CSF2-Ab level | Average | 4,450 | 3,410 |
|  | SD | 6,546 | 3,648 |
| *P* value (vs BMI <25) | |  | 0.207 |
|  |  |  |  |
| Complication | | Cardiovascular events+ | Cardiovascular events- |
| Sample number | | 7 | 237 |
| s-CSF2-Ab level | Average | 5,851 | 4,089 |
|  | SD | 3,157 | 5,822 |
| *P* value (vs cardiovascular events-) | | | **0.034** |
|  |  |  |  |
| Complication | | Cerebrovascular events+ | Cerebrovascular events- |
| Sample number | | 6 | 237 |
| s-CSF2-Ab level | Average | 4,339 | 4,089 |
|  | SD | 3,167 | 5,822 |
| *P* value (vs Cerebrovascular events-) | | | 0.316 |
|  |  |  |  |
| Complication | | Cancer+ | Cancer- |
| Sample number | | 26 | 237 |
| s-CSF2-Ab level | Average | 4,344 | 4,089 |
|  | SD | 5,404 | 5,822 |
| *P* value (vs cancer-) | | | 0.856 |

The participants were divided into two categories according to the following classifications: age (≥65 versus <65 years), sex (male versus female), cardio-cerebral vascular events, cardiovascular events, cerebrovascular events, and cancer. The Mann–Whitney *U* test was employed to compare the s-CSF2-Ab levels between the two groups. The sample numbers, averages, standard deviations of counts, and *P* values are presented. Significant correlations (*P* <0.05) are marked in bold. s-CSF2-Ab, serum CSF2 antibody

**Supplementary Table 2.** Baseline characteristics of the study patients

|  |  | HD | AIS | TIA |
| --- | --- | --- | --- | --- |
| Total number | | 109 | 196 | 79 |
| Age (years) | | 59.82 ± 7.90 | 75.13 ± 7.28 | 70.67 ± 12.75 |
| Sex (male) |  | 62 (56.9%) | 122 (62.2%) | 46 (58.2%) |
| Hypertension | | 34 (31.2%) | 153 (78.1) | 53 (67.0%) |
| Diabetes |  | 9 (8.3%) | 53 (27.0%) | 19 (24.1%) |
| Hyperlipidemia | | 31 (28.4%) | 56 (28.6%) | 30 (40.0%) |
| CVD |  | 1 (0.9%) | 10 (5.1%) | 4 (5.1%) |
| Obesity (BMI ≥25) | | 37 (33.9%) | 56 (28.6%) | 28 (35.4%) |
| Smoking |  | 47 (43.1%) | 107 (54.6%) | 29 (31.6%) |

AIS, acute ischemic stroke; TIA, transient ischemic attack; HD, healthy donor; CVD, cardiovascular disease; BMI, body mass index.

**Supplementary Table 3.** Correlation analysis of the s-CSF2-Ab levels with data of the patients in the CKD cohort

|  |  | s-CSF2-Ab |
| --- | --- | --- |
|  | *r* value | *P* value |
| Age | 0.0906 | 0.1176 |
| Height | -0.0233 | 0.6878 |
| Plaque score | 0.1902 | **0.0010** |
| Max-IMT | 0.1201 | **0.0389** |
| ABI (right) | -0.0574 | 0.3286 |
| ABI (left) | -0.0318 | 0.5870 |
| CAVI (right) | 0.1383 | **0.0204** |
| CAVI (left) | 0.1214 | **0.0412** |
| HbA1c | -0.0461 | 0.5783 |
| Fe | -0.1237 | **0.0322** |
| Ferritin | 0.0588 | 0.3104 |
| TSAT ratio | -0.0874 | 0.1310 |
| Kt/V | -0.0762 | 0.1882 |
| RBC | -0.0495 | 0.3932 |
| HGB | -0.0922 | 0.1111 |
| HCT | -0.0288 | 0.6190 |
| PLT | -0.0835 | 0.1489 |
| TP | -0.0372 | 0.5212 |
| Albumin | -0.1344 | **0.0199** |
| UN | -0.1109 | 0.0551 |
| CRE | -0.0762 | 0.1881 |
| UA | -0.0353 | 0.5426 |
| Na | 0.0576 | 0.3205 |
| K | 0.0238 | 0.6810 |
| Cl | 0.0534 | 0.3563 |
| Ca | 0.0314 | 0.5874 |
| P(inorganic) | 0.0313 | 0.5895 |
| Ca (corrected) | 0.0940 | 0.1041 |
| Mg | 0.0449 | 0.4382 |
| AST | 0.1623 | **0.0048** |
| ALT | 0.0885 | 0.1260 |
| LDH | 0.1796 | 0.0018 |
| gamma-GTP | 0.0807 | 0.1635 |
| ALP | 0.0764 | 0.1872 |
| tBil | -0.0056 | 0.9228 |
| AMY | -0.0240 | 0.6789 |
| CK | -0.0228 | 0.6944 |
| Total-Chol | -0.0243 | 0.6754 |
| HDL-C | -0.0934 | 0.1064 |
| LDL-C | 0.0111 | 0.8479 |
| TG | 0.0174 | 0.7643 |
| CRP | 0.1746 | **0.0024** |

Correlation coefficients (*r* values) and *P* values obtained using Spearman’s rank-order correlation analysis are shown.

The following patient data were included: age, height, plaque score, maximum intima–media thickness (max-IMT), CAVI (cardio-ankle vascular index), ABI (ankle brachial pressure index), glycated hemoglobin (HbA1c), iron (Fe), ferritin, transferrin saturation ratio (TSAT ratio), standardized urea clearance (Kt/V), red blood cell number (RBC), hemoglobin (HGB), hematocrit (HCT), platelet number (PLT), total protein (TP), albumin, urea nitrogen (UN), creatinine (CRE), uric acid (UA), sodium (Na), potassium (K), chloride (Cl), calcium (Ca), inorganic phosphate (IP), magnesium (Mg), aspartate aminotransferase (AST), alanine amino transferase (ALT), lactate dehydrogenase (LDH), gamma-glutamyl transpeptidase (gamma-GTP), alkaline phosphatase (ALP), total bilirubin (tBil), amylase (AMY), creatinine kinase (CK), total cholesterol (T-CHO), high-density lipoprotein cholesterol (HDL-C), low-density lipoprotein cholesterol (LDL-C), triglyceride (TG), and C-reactive protein (CRP). Significant correlations (*P* < 0.05) are marked in bold.

**Supplementary Table 4.** Logistic regression analysis of the predictive factors for AIS (total no, 384; number of events, 196)

| Univariate analysis | | | Multivariate analysis | | |
| --- | --- | --- | --- | --- | --- |
|  | *P* value |  | OR | 95% CI | *P* value |
| Age, years (≥60) | **<0.0001** |  | 12.2 | 5.20-28.70 | **<0.0001** |
| Male | 0.350 |  |  |  |  |
| hypertension | **<0.0001** |  | 2.77 | 1.64-4.68 | **<0.0001** |
| DM | **0.004** |  | 1.79 | 0.96-3.34 | 0.068 |
| Lipidemia | 0.439 |  |  |  |  |
| CVD | 0.294 |  |  |  |  |
| Obesity (BMI ≥25) | 0.227 |  |  |  |  |
| Smoking | **0.049** |  | 1.34 | 0.77-2.34 | **0.038** |
| CSF2-Ab (≥5102) | **0.010** |  | 1.73 | 1.07-2.8 | **0.026** |

Significant associations (*P* <0.05) are marked in bold. The CSF2-Ab cutoff value was 5102 based on the receiver operating characteristic curve analysis. OR, odds ratio; CI, confidence interval

**Supplementary Table 5.** Comparison of serum CSF2 antibody levels according to stage and age of the patients with CRC.

|  | s-CSF2-Ab+ | s-CSF2-Ab- |
| --- | --- | --- |
| age ≥65 | 36 | 31 |
| age <65 | 17 | 29 |
| *P* value |  | 0.0792 |
|  |  |  |
| Stage III and IV | 21 | 26 |
| Stage 0, I and II | 32 | 34 |
| *P* value |  | 0.6896 |

According to the Japanese Classification of Colorectal, Appendiceal, and Anal Carcinoma: the 3d English Edition (Secondary Publication), the number of patients for colorectal cancer was as follows: 5 patients in stage 0, 29 in stage I, 32 in stage II, 31 in stage III, and 16 in stage IV. Chi-square tests were performed.

**Supplementary Figure 1.** Full blot images of Figure 1.


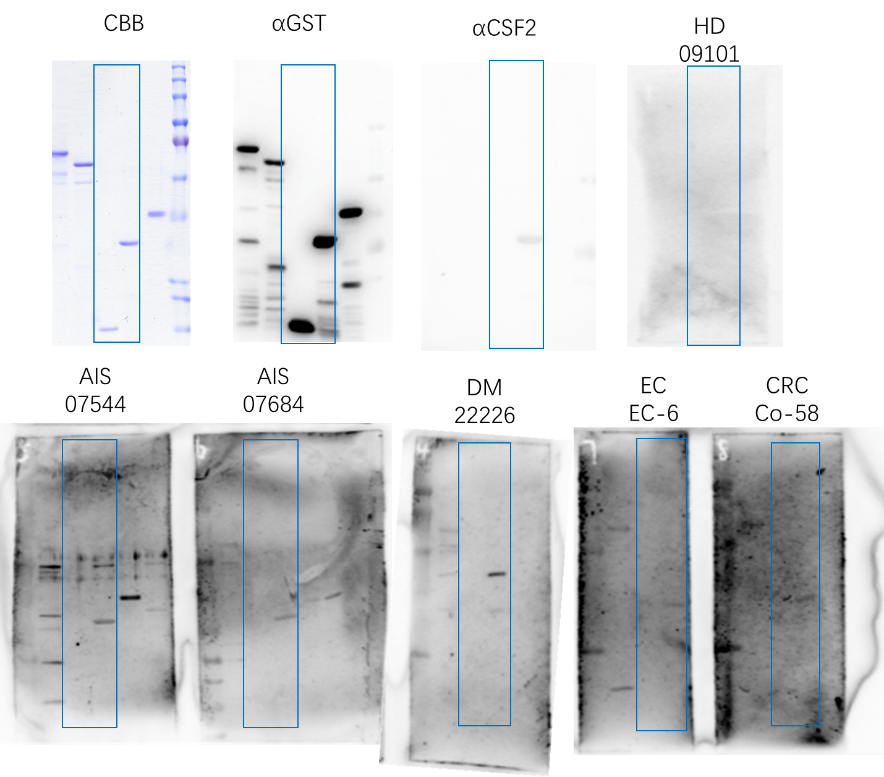


The parts enclosed by the square of CBB, αGST, αCSF2, AIS-07544, AIS-07684, DM-22226, EC-6, Co-58, HD-09101 correspond to CBB, αGST, αCSF2, AIS-07544, AIS-07684, DM-22226, EC-6, Co-58, HD-09101, respectively, of Figure 1.

**Supplementary Figure 2.** Detection of serum antibodies against CSF2


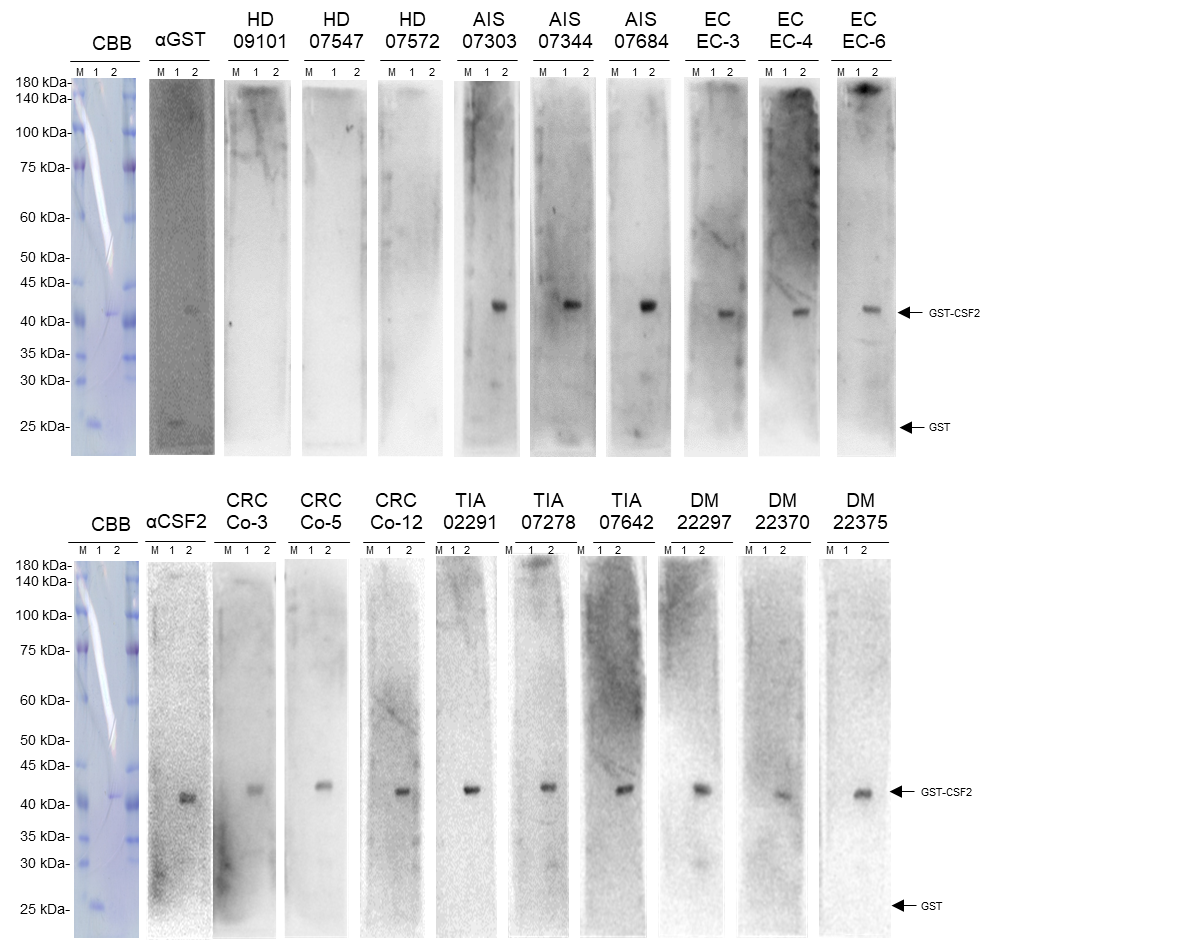


Presence of antibodies against CSF2 in the serum samples of patients with acute ischemic stroke (AIS), diabetes mellitus (DM), esophageal cancer (EC), and colorectal cancer (CRC). Purified GST-CSF2 (lane 1) and GST (lane 2) proteins were separated on sodium dodecyl sulfate–polyacrylamide gels, followed by staining with Coomassie Brilliant Blue (CBB) or western blotting using anti-GST (αGST), anti-CSF2 (αCSF2), and serum IgG antibodies from patients with AIS (#007303, #07344, and #07684), TIA (#02291, #07278, and #07642), DM (#22297, #22370, and #22375), EC (#EC-3, #EC-4, and #EC-6), and CRC (#Co-3, #Co-5, and #Co-12) but not by the serum of the HD (#09101, #07547, and #07572). Molecular weight markers are presented in the left lane. Arrows indicate protein sizes: GST-CSF2, 44.6 kDa; GST, 26 kDa. CSF2, colony-stimulating factor 2

**Supplementary Figure 3.** Survival analysis of patients with esophageal cancer and gastric cancer


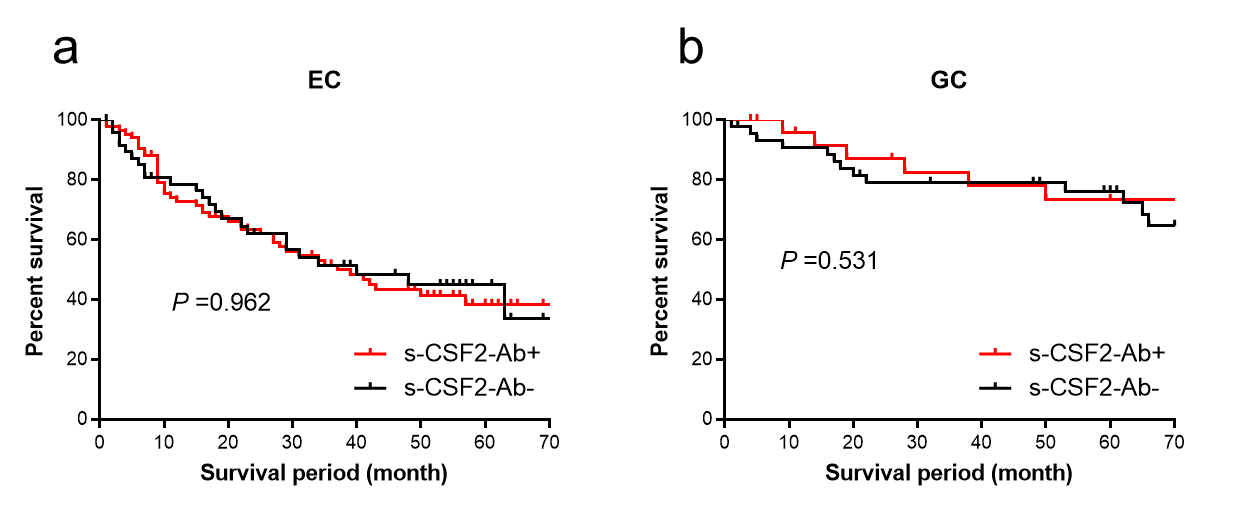


The levels of s-CSF2-Ab in healthy donors (HDs) and patients with esophageal cancer (EC) and gastric cancer (GC) were examined *via* amplified luminescence proximity homogeneous assay-linked immunosorbent assay (AlphaLISA). The cutoff values in patients with EC and GC were determined using receiver operating characteristic curve analysis (Figure 5b and 5c). (**a**) The overall postoperative survival rates of patients with EC were compared according to the s-CSF2-Ab status (*P* = 0.962). (**b**) The overall survival rates of patients with GC were compared according to the s-CSF2-Ab status (*P* = 0.531). Kaplan–Meier with the log-rank test

**Supplementary Figure 4.** MTS Assay


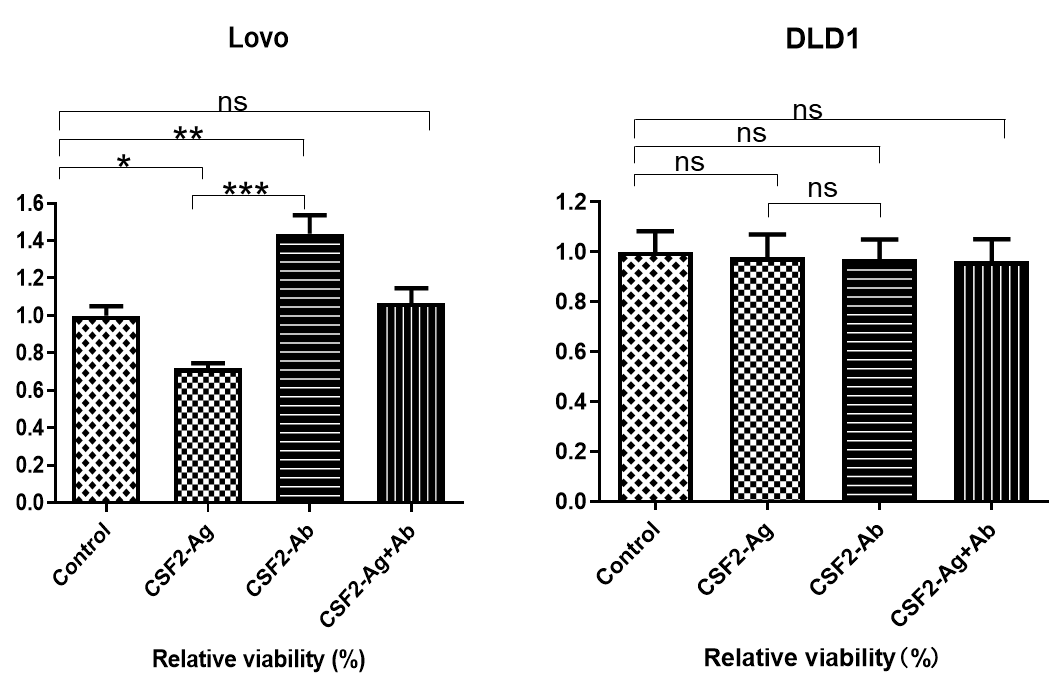


In LoVo cells, treatment with CSF2 for 72 h led to a reduction in cell viability, whereas treatment with the anti-CSF2 antibody led to an increase in cell viability. No significant changes in cell viability were observed under the same conditions in DLD1 cells expressing mutant p53. Error bars represent the standard deviations. ** *P* <0.01 versus control, repeated-measured ANOVA with Tukey's post hoc comparison test.

MTS, 3-(4,5-dimethylthiazol-2-yl)-5-(3-carboxymethoxyphenyl)-2-(4-sulfophenyl)-2H-tetrazolium

**Supplementary Figure 5.** Luciferase reporter assay


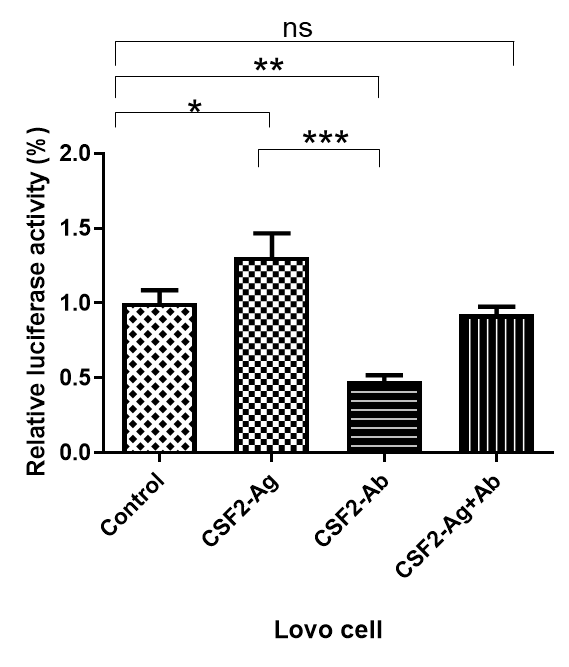


The p21-Luc reporter, which was preactivated by the cotransfection with wild-type p53, was further activated by the CSF2 treatment and was significantly inactivated by treatment with the anti-CSF2 antibody (*P* <0.01). The decrease in the activation of p21-Luc by the anti-CSF2 antibody was reversed by the concurrent addition of excess antigenic CSF2. The LoVo cells (1 × 10^5^ cells/well) seeded on 24-well plates were transfected with a p53 expression plasmid plus firefly luciferase (p21-Luc) and control *Renilla* luciferase reporter plasmid, SV40-Rluc, using Lipofectamine Plus. After 24 h, 10-μg/mL anti-CSF2 antibody and/or 1-μg/mL CSF2 were added. Two days after the transfection, firefly and *Renilla* luciferase activities were determined using a Dual-Luciferase Assay System and a luminescence reader. Firefly luciferase activity was normalized to the *Renilla* luciferase control activity. Error bars represent the standard deviations. ** *P* <0.01 vs control, repeated-measured ANOVA with Tukey's post hoc comparison test.

**Supplementary Figure 6.** Analysis of Ser-46-phosphorylated p53 levels in the LoVo cells treated with the CSF2 or anti-CSF2 antibody


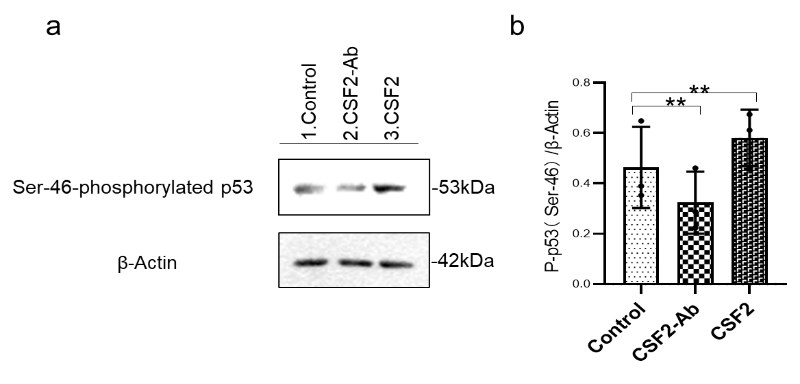


The expression levels of Ser-46-phosphorylated p53 decreased after treatment with the anti-CSF2-antibody and increased after treatment with CSF2. The LoVo cells were incubated with 1 µg/mL of CSF2 or 10 µg/mL of anti-CSF2 antibody for 4 h, and the expression levels of phosphorylated p53 and the control protein β-actin were analyzed using western blotting (**a**). This experiment was independently repeated three times, and the relative intensities of the bands were quantified using the ImageJ software (**b**). Error bars represent the standard deviations. ** *P* <0.01 vs control, two-sided Student’s *t*-test
